# The *Corynebacterium pseudotuberculosis in silico *predicted pan-exoproteome

**DOI:** 10.1186/1471-2164-13-S5-S6

**Published:** 2012-10-19

**Authors:** Anderson R Santos, Adriana Carneiro, Alfonso Gala-García, Anne Pinto, Debmalya Barh, Eudes Barbosa, Flávia Aburjaile, Fernanda Dorella, Flávia Rocha, Luis Guimarães, Meritxell Zurita-Turk, Rommel Ramos, Sintia Almeida, Siomar Soares, Ulisses Pereira, Vinícius C Abreu, Artur Silva, Anderson Miyoshi, Vasco Azevedo

**Affiliations:** 1 Molecular and Celular Genetics Laboratory, Instituto de Ciências Biológicas, Universidade Federal de Minas Gerais, Belo Horizonte, Minas Gerais, Brazil; 2 DNA Polimorfism Laboratory, Universidade Federal do Pará, Campus do Guamá - Belém, PA, Brazil; 3 Centre for Genomics and Applied Gene Technology, Institute of Integrative Omics and Applied Biotechnology, Nonakuri, Purba Medinipur, West Bengal, India

## Abstract

**Background:**

Pan-genomic studies aim, for instance, at defining the core, dispensable and unique genes within a species. A pan-genomics study for vaccine design tries to assess the best candidates for a vaccine against a specific pathogen. In this context, rather than studying genes predicted to be exported in a single genome, with pan-genomics it is possible to study genes present in different strains within the same species, such as virulence factors. The target organism of this pan-genomic work here presented is *Corynebacterium pseudotuberculosis*, the etiologic agent of caseous lymphadenitis (CLA) in goat and sheep, which causes significant economic losses in those herds around the world. Currently, only a few antigens against CLA are known as being the basis of commercial and still ineffective vaccines. In this regard, the here presented work analyses, *in silico*, five *C. pseudotuberculosis *genomes and gathers data to predict common exported proteins in all five genomes. These candidates were also compared to two recent *C. pseudotuberculosis in vitro *exoproteome results.

**Results:**

The complete genome of five *C. pseudotuberculosis *strains (1002, C231, I19, FRC41 and PAT10) were submitted to pan-genomics analysis, yielding 306, 59 and 12 gene sets, respectively, representing the core, dispensable and unique *in silico *predicted exported pan-genomes. These sets bear 150 genes classified as secreted (SEC) and 227 as potentially surface exposed (PSE). Our findings suggest that the main *C. pseudotuberculosis in vitro *exoproteome could be greater, appended by a fraction of the 35 proteins formerly predicted as making part of the variant *in vitro *exoproteome. These genomes were manually curated for correct methionine initiation and redeposited with a total of 1885 homogenized genes.

**Conclusions:**

The *in silico *prediction of exported proteins has allowed to define a list of putative vaccine candidate genes present in all five complete *C. pseudotuberculosis *genomes. Moreover, it has also been possible to define the *in silico *predicted dispensable and unique *C. pseudotuberculosis *exported proteins. These results provide *in silico *evidence to further guide experiments in the areas of vaccines, diagnosis and drugs. The work here presented is the first whole *C. pseudotuberculosis in silico *predicted pan-exoproteome completed till today.

## Background

Reverse Vaccinology (RV) [1] analyses the genome sequence of a pathogen, which is an expected coded sequence for all the possible expressed genes in the pathogen's life cycle. All Open Reading Frames (ORF's) derived from the genome sequence can be evaluated using a computer program to determine their ability as vaccine candidates, giving special attention to exported proteins, as these are essential in host-pathogen interactions. Examples of such interactions include: (i) adherence to host cells, (ii) invasion of the cell to which there is compliance, (iii) damage to host tissues, (iv) environmental stresses resistance from the defense machinery of the cell being infected, and (v) mechanisms for subversion of host immune response [2-5].

Regarding exported proteins, these can distinguish between those that are exported to the cell wall, and after cleaved, release the mature portion into the extracellular milieu, which are referred to as secreted proteins (SEC), and those proteins exported to the cell wall which, even after cleaved, do not release the mature portion to the extracellular milieu, due to one or more hydrophobic motifs causing anchoring to the cell wall, and which are referred to as potentially surface exposed proteins (PSE). Different PSE subcategories exist according to the presence of a carboxy (C) or amino (N) terminal portion anchored to the cell wall, lipoproteins (E), end terminal loops (L), retention signals-like such as LGxTG, LysM, GW, Choline binding and PG binding (R), in combination or not with other PSE subcategories [6].

The term 'Reverse' from RV can be explained by the reverse genetics (RG) technique. Before the dawn of genomic, there were attempts to discover the responsible genes from a phenotype, reversing the research path of Crick's Central Dogma [7] (DNA → RNA → Protein) discovery. Holding the likely gene sequence, several techniques can be used to identify gene sequence modifications responsible for changes in the organism's phenotype. Crick's Central Dogma principle is also used for RV, as this technique searches within a gene sequence for possible proteins that could act as antigens capable of stimulating an immune response in a host organism [8].

The concept of RV was adapted to fit a new reality of widespread availability of genomic data [9]. With this technique, instead of searching for targets in a single strain or subspecies of an organism, it is now possible to simultaneously research in dozen of genomes, exploring potential joint antigens or exclusive ones to multiple genomes [10]. The availability of a large number of genomes to implement RV has lead to the emergence of the pan-genomics reverse vaccinology concept [11], which can also apply to the concepts of core, extended (dispensable) and character (unique) genomes. While the core genome is composed of exported genes (genes that transcribe for exported proteins) that are common to these multiple strains and could represent candidates for a vaccine, the dispensable genome consists of genes that are absent in at least one of the strains of the studied species and the unique genome consists of genes that are specific to only a particular a strain [10]. From the standpoint of vaccines, the core genome represents to be a good candidate to compose a vaccine that is suitable for all studied strains. In this regard, the first step to enable any pan-genomic reverse vaccinology study is to predict the core genome, along this work denominated *in silico *predicted pan-exoproteome (ISPPE). The model organism here analyzed *(C. pseudotuberculosis) *is a Gram-positive (GRAM+) bacterium, intracellular facultative parasite that affects small ruminants causing a chronic infectious pyogranulomatous disease characterized by the formation of abscesses in lymph nodes [12]. This pathogen infects mainly goats and sheep causing caseous lymphadenitis, but can also infect a huge variety of hosts throughout the world such as camels, horses, cattle, buffaloes, llamas, alpacas and, more rarely, humans [13-18], causing different diseases with different degrees of severity in each of them [12,19].

## Results and discussion

### In silico exoproteome prediction schema

**Figure 1:**
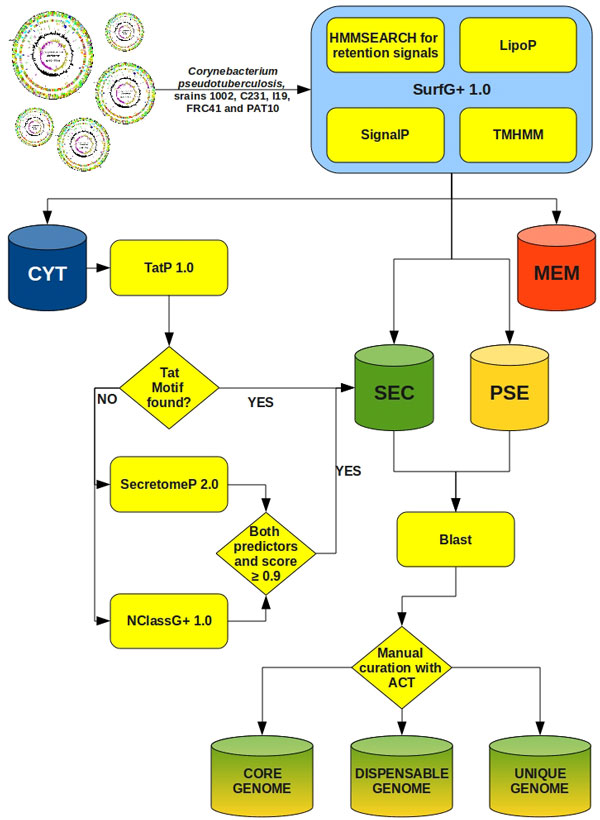
***C. pseudotuberculosis *pan genomic prediction schema**. Software used, identified sub-cellular compartments and flow scheme to create the final pan genomic data sets.

**Figure 2:**
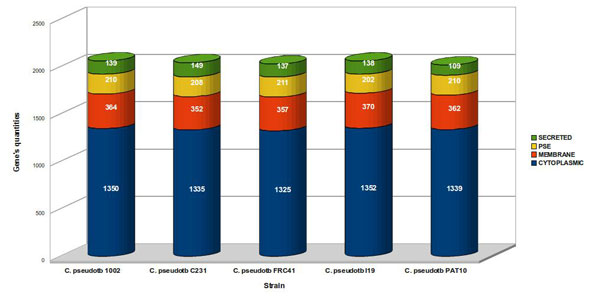
**Predicted gene quantities by sub-cellular compartment from full *C. pseudotuberculosis *genomes**. Classification of more than 10,000 distinct genes from the five different *C. pseudotuberculosis *strains in the four sub-cellular categories: cytoplasmic (CYT), membrane (MEM), potentially surface exposed (PSE) and secreted (SEC). Predictions were made using the schema presented in Figure 1.

As shown in our proposed prediction schema (Figure 1), the software SurfG+ (Surface Gram positive), specially configured for GRAM+ bacteria, is responsible for most of the sub-cellular classifications, which vary between cytoplasmic (CYT), membrane (MEM), SEC and PSE (Figure 2). SurfG+ was configured for GRAM+ bacteria. Figure 1 represents the prediction schema using SurfG+ and three additional software, TatP 1.0 [20], SecretomeP 2.0 [21] and NclassG+ [22], which are specialized in non-classical secretion prediction. SurfG+ incorporates SignalP 3.0 predictor, responsible for identification of classical putative secreted proteins or exported proteins by the SEC pathway [23].

The results obtained after running SurfG+, TapP, SecretomeP and NClassG+ have gave rise to two gene data sets labeled as SEC and PSE, which correspond to the *C. pseudotuberculosis *ISPPE. These ISPPE data sets are composed of putative proteins present fivefold (5x), fourfold (4x), threefold (3x), twofold (2x) or onefold (1x), where fivefold means that a gene was predicted in all five strains, four fold meaning that a gene was predicted in four strains, and so on. A gene fold was obtained by reciprocal blast results, as described in the methods section. Since not all predicted genes are named, it was necessary to create a pan genome identifier, here denominated pan *locus*, to nominate each unique gene fold. The pan *locus *is unique within a pan genome and is shared by all homologous genes. For example, when a putative exported protein was found within the five strains, each gene copy received the same pan *locus *to facilitate further data processing and identification. Following, it was necessary to confirm these results by systematical manual curation of each gene using the ACT tool from the Artemis software package [24]. Once completed this manual curation, it was possible to answer several questions regarding the correctness of each blast result and, as a consequence, it was possible to identify, for instance, that a gene formerly classified as 1x was indeed a 5x, as the other four gene copies were created starting beyond the signal peptide motif. After initial methionine correction, and also taking into account homologous genes, a new prediction step indicated all remaining putative proteins to be exported, composing the core ISPPE. However, gene's start positions incorporating a less probable signal peptide motif were also observed. In general, genes formerly predicted as Nx proved to be correct by manual curation as the remaining (5-N)x genes were predicted as cytoplasmic, PSE or pseudogenes. These results are particularly interesting because they compose the dispensable and unique ISPPE data sets. These genome annotation corrections, as a consequence of these analyses, were incorporated into the official annotation of the five *C. pseudotuberculosis *strains deposited at GenBank in August, 2011. This genomes are also available in the additional file 1, as EMBL files.

### Classical and non-classical secreted putative proteins

**Figure 3:**
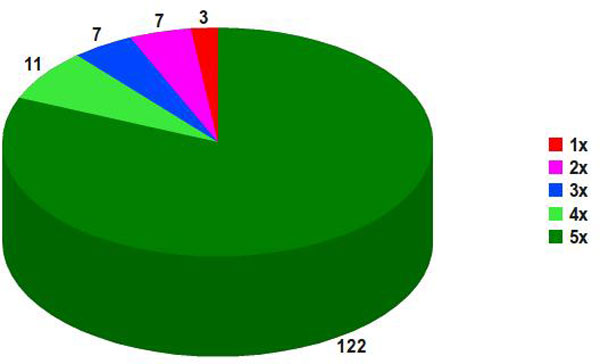
**Predicted *C. pseudotuberculosis *pan secretome**. Predictions for 150 genes from strains 1002, C231, I19, FRC41 and PAT10 made by SurfG+ 1.0, TatP 1.0 Server and SecretomeP 2.0 Server.

**Figure 4:**
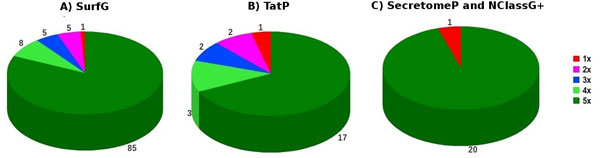
**Predicted *C. pseudotuberculosis *pan secretome by predictor software**. Predicted secreted genes coverage in the predicted pan secretome of the five bacterial strains separated by predictor software SurfG+, TatP and SecretomeP.

Figure 3 exhibits the *in silico *predicted pan secretome results for *C. pseudotuberculosis*, which comprise 150 genes, out of 377 from the whole ISPPE, representing 750 *locus_tags *in the five studied *C. pseudotuberculosis *strains. However, despite representing 750 *locus_tags*, not all were predicted as secreted. If at least one gene copy, within a specific pan *locus*, was not predicted as secreted, it still received the same pan *locus *but was not classified as part of the predicted core secretome. There are 122 genes composing the predicted core secretome (5x), followed by 25 genes constituting the predicted dispensable secretome (4x, 3x and 2x) and just 3 genes as the predicted unique secretome (1x). These results were obtained applying the prediction schema from Figure 1; however, different contributions were obtained from different predictors, as shown in Figure 4.

SurfG+ predicted 104 genes, corresponding 85, 18 and 1 to the predicted core, dispensable and unique secretome respectively. On the other hand, TatP predicted 25 genes, of which 17, 7 and 1 corresponded to the predicted core, dispensable and unique secretome respectively. Finally, SecretomeP and NClassG+ predicted 21 genes, corresponding 20 and 1 to the predicted core and unique secretome respectively. It can be easily observed that the main predicted portion is originated by SurfG+, as it predicts putative proteins possibly secreted by the SEC pathway. A considerable portion of genes (~31%), only within the predicted core secretome, comes from non-classical secretion predictors that cannot be ignored when the subject is about vaccine candidates.

The dispensable and unique *C. pseudotuberculosis *predicted secretomes contain ~8%, or 58 *locus_tags*, not predicted as secreted. Putative proteins predicted as CYT, PSE and putative frame shifts (pseudogenes) account for 22, 24 and 10 *locus_tags *respectively. In the dispensable and unique *C. pseudotuberculosis in silico *predicted secretomes, the numbers of genes identified as membrane integral or absent in a genome are insignificant. Nevertheless, the manual curation step ensured no annotation errors in these predictions, making it possible to claim the hypothesis that these differences could be due to environment adaptations. A table containing the complete list of *C. pseudotuberculosis *secreted proteins is available in the additional file 2.

### Potentially surface exposed (PSE) putative proteins

**Figure 5:**
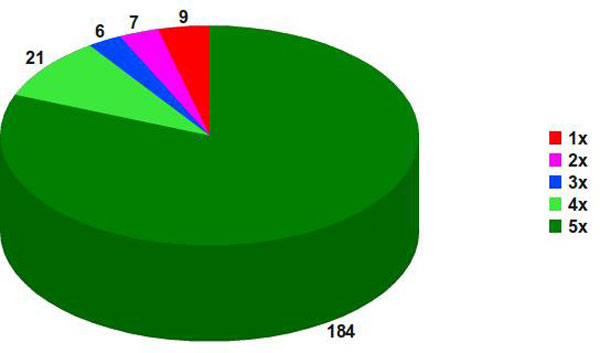
**Predicted *C. pseudotuberculosis *pan surfaceome**. Pan surfaceome predictions for 227 genes from strains 1002, C231, I19, FRC41 and PAT10, performed by SurfG+ 1.0.

The SurfG+ software was calibrated by the cell wall thickness for each *C. pseudotuberculosis *strain. Figure 5 shows 184 genes, out of 377 from the whole ISPPE, comprising the predicted core surfaceome (5x), 34 genes composing the predicted dispensable surfaceome (4x, 3x and 2x) and just 9 genes as predicted unique surfaceome (1x). These 227 genes account for 1135 *locus_tags *in all five strains. In this set, homologous genes within a pan *locus *do not ever share the same sub-cellular prediction. Genes predicted as MEM, CYT, SEC and putative pseudogenes account for 29, 23, 20 and 17 distinct *locus_tags*, respectively. Genes predicted as MEM (~3%) compose the second major group. This could be explained by the fact that membrane proteins already contain hydrophobic extension and could be more susceptible to expose or occult parts of a protein to the extracellular milieu. However, the same reasoning does not suit to explain the third major group of *locus_tags *with surfaceome pan *locus *that correspond to proteins predicted as secreted ones. These 20 *locus_tags *that were predicted as secreted, but also received surfaceome pan *locus*, raise a question; do these fit SEC or PSE labels? There exist no simple paths to estimate their sub-cellular compartment by software, since some *locus_tags *were predicted as PSE receiving surfaceome pan *locus *and other were predicted as SEC and also received secretome pan *locus*. Ten pan *locus *(plcppse193, plcppse194, plcppse205, plcppse218, plcppse226, plcpsec096, plcpsec097, plcpsec098, plcpsec100, plcpsec101) faces this question, as some genes appear in both the predicted secretome and surfaceome.

**Figure 6:**
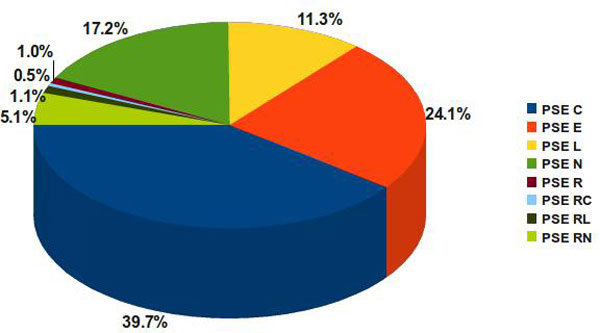
**Predicted *C. pseudotuberculosis *pan surfaceome by PSE subcategories**. PSE categories are distributed in outward C-terminal or N-terminal portion greater than or equal 50 AA. Outward N or C terminal greater than 100 AA are classified as L. Lipogenes identified by LipoP are classified as E and retention signals identified by HMMSEARCH profiles are classified as R. These labels can also be conjugated to create other PSE subcategories.

The PSE subcategories show predominance of genes, as presented in Figure 6. Most of the 1045 genes predicted as PSE are cell wall anchored outward C-terminal (~40%) (≥ 50 AA long), followed by lipoproteins (~24%), outward loops (~11%) (≥ 100 AA long) and outward N-terminal (~17%) (≥ 50 AA long), whereas genes containing retention signals (PSE R) account only for ~8%.

The PSE results of all strains were analyzed considering that a significant cell wall thickness difference between strain I19 and the other ones was observed (~34 nm versus ~24 nm). Despite the significant cell wall thickness difference, a small difference was predicted in the genome, which accounts for a decrease in the number of PSE and an increase in the number of MEM genes in *C. pseudotuberculosis *strain I19. A table containing the complete list of *C. pseudotuberculosis *PSE proteins is available in the additional file 3.

### Revised *in vitro *exoproteome results

The 104 observed genes in both TPP/LC-MS^E ^[25] and 2-DE-MALDI-TOF/TOF, (Silva WM, Seyffert N, Castro TLP, Santos AV, Pacheco LGC, Santos AR, Ciprandi A, Zurita-Turk M, Dorella FA, Andrade HM, Pimenta AMC, Silva A, Miyoshi A, Azevedo V, unpublished observations) experiments were compared with the ISPPE results here presented. This comparison, explained in the methods section, brought novel insights into the *in vitro *exoproteome and showed the possibility of having additional genes in the main *C. pseudotuberculosis in vitro *exoproteome. In Table 1 are listed all 35 proteins of the variant *in vitro *exoproteome (strains 1002 and C231), that correspond to ~23% of the total amount. These proteins were found to be highly conserved in the five compared *C. pseudotuberculosis *strains and comprise the core ISPPE. Moreover, it was verified that three proteins (ADL20466, ADL20097 e ADL19973), previously classified as belonging to the variant *in vitro *exoproteome of strains 1002 [25], did actually belong to the main *in vitro *exoproteome. These findings give raise to the possibility that more proteins of the variant *in vitro *exoproteome indeed make part of the main *in vitro *exoproteome.

**Table 1:** Core *C. pseudotuberculosis in silico *predicted pan-exoproteome found in the variant *in vitro *exoproteome

Protein identifier	*locus_tag*	Gene name	Product	Predicted local sub-cellular	GenBank organism identifier
ADL19972	Cp1002_0064		Hypothetical protein	PSE E	CP001809

ADL20140	Cp1002_0237	*slpA*	Surface layer protein A	SEC	CP001809

ADL20222	Cp1002_0320		Hypothetical protein	PSE N	CP001809

ADL20288	Cp1002_0388		L,D-transpeptidase catalytic domain, region YkuD	SEC	CP001809

ADL20391	Cp1002_0497	*malE*	Maltose/maltodextrin transport system substrate-binding protein	PSE E	CP001809

ADL20455	Cp1002_0562	*sprT*	Trypsin	PSE C	CP001809

ADL20477	Cp1002_0584	*cynT*	Carbonic anhydrase	PSE E	CP001809

ADL20508	Cp1002_0615		Hypothetical protein	SEC	CP001809

ADL20574	Cp1002_0681	*rpfB*	Resuscitation-promoting factor RpfB	SEC	CP001809

ADL20656	Cp1002_0766		Hypothetical protein	SEC	CP001809

ADL21028	Cp1002_1144	*yceG*	Amino deoxychorismate lyase	SEC	CP001809

ADL21239	Cp1002_1362		Hypothetical protein	PSE E	CP001809

ADL21302	Cp1002_1425	*ctaC*	Cytochrome c oxidase subunit II	PSE C	CP001809

ADL21537	Cp1002_1669		Hypothetical protein	SEC	CP001809

ADL21667	Cp1002_1802	*lipY*	Secretory lipase	SEC	CP001809

ADL09524	CpC231_0025	*pld*	Phospholipase D	SEC	CP001829

ADL09532	CpC231_0033	*pbpA*	Penicillin-binding protein A	SEC	CP001829

ADL09691	CpC231_0196		Hypothetical protein	SEC	CP001829

ADL09697	CpC231_0203	*pbpB*	Penicillin binding protein transpeptidase	SEC	CP001829

ADL09852	CpC231_0360	*oppA1*	Oligopeptide-binding protein oppA	PSE E	CP001829

ADL09871	CpC231_0379		Hypothetical protein	SEC	CP001829

ADL09872	CpC231_0380	*malE*	Maltotriose-binding protein	PSE E	CP001829

ADL09990	CpC231_0503	*lytR*	Transcriptional regulator lytR	PSE C	CP001829

ADL10248	CpC231_0766		Hypothetical protein	SEC	CP001829

ADL10460	CpC231_0982	*ciuA*	Iron ABC transporter substrate-binding	PSE E	CP001829

ADL10489	CpC231_1012	*yceI*	Protein yceI	SEC	CP001829

ADL10626	CpC231_1150		Zinc metallopeptidase	PSE C	CP001829

ADL10663	CpC231_1187		Lipoprotein	PSE E	CP001829

ADL10880	CpC231_1409	*pknL*	Serine/threonine protein kinase	PSE N	CP001829

ADL11196	CpC231_1737		Corynomycolyl transferase	SEC	CP001829

ADL11213	CpC231_1756		Hypothetical protein	SEC	CP001829

ADL11326	CpC231_1871		Hypothetical protein	PSE N	CP001829

ADL11338	CpC231_1885		Membrane protein	SEC	CP001829

ADL11339	CpC231_1886		Hypothetical protein	SEC	CP001829

ADL11410	CpC231_1959	*glpQ*	Glycerophosphoryl diester phosphodiesterase	PSE E	CP001829

The 35 proteins listed in this table were not found in the experimental main *in vitro *exoproteome [47; 48] but were found in the *in silico *predicted pan-exoproteome of all five *C. pseudotuberculosis *strains.

This comparison also served as a rebuttal argument against some specific genes. The Cp1002_0369 gene, classified under the plcpsec100 pan *locus *as a pseudogene, was identified by the *in vitro *exoproteome experiment. Interestingly, this gene copy also suits the plcppse226 pan *locus*. Both pan *locus *make part of previous related genes that already showed difficulties to be classified, by software, into any potential sub-cellular compartment, as some genes within the pan *locus *fit both SEC and PSE labels. The *in silico *predictions enforces that there are at least three secreted proteins, inspite of the other two gene copies being predicted as having PSE and CYT labels.

Furthermore, the genes plcppse180, plcppse192, plcpsec077, plcpsec095 and plcpsec099 also had both genes found in the main *in vitro *exoproteome of strains 1002 and C231, but were not classified in the ISPPE. The plcppse180 pan *locus *holds a putative pseudogene (CpPAT10_0459), and is therefore not present in the *in silico *predicted core surfaceome. Other genes were predicted as cytoplasmic. It is possible that these genes were wrongly assembled since there is evidence that at least two homologous genes, from strains 1002 and C231, are exported to the extracellular milieu.

### Core *C. pseudotuberculosis *ISPPE candidates homologous to *Mtb*

Within the core *C. pseudotuberculosis *ISPPE, homologous genes to those of the previously studied *Mycobacterium tuberculosis *H37Rv (*Mtb*) were observed. In this work we present some of these homologous genes featuring at least 90% protein alignment and 50% identity within this alignment. These cut-offs were obtained during the search for *C. pseudotuberculosis *homologous genes in the *Mtb *genome.

The core *C. pseudotuberculosis *ISPPE, that accounts for ~81% of the total, is composed of 306 genes or 1,530 distinct *locus_tags*, being ~40% predicted as SEC and ~60% predicted as PSE proteins, of which 20 genes present high similarity to *Mtb*'s genes (Table 2); however, not all of these *Mtb *genes have known functions.

**Table 2:** Core *C. pseudotuberculosi s in silic o *predicted pan-exoproteome homologous to *Mtb*'s proteins

*Corynebacterium pseudotberculosis*				*Mycobacterium* *tuberculosis*				
**pan *locus***	**Reference genome *locus_tag***	**ORF size**	**% of amino acid alignment's identity**	**ORF size**	***locus_tag***	**Gene name**	**protein ID**	**Annotated product**

plcpsec106	cpfrc_00104	488	69.10	461	Rv3790		NP_218307.1	oxidoreductase

plcpsec076	cpfrc_00276	371	51.56	357	Rv2678c	*hemE*	NP_217194.1	uroporphyrinogen decarboxylase

plcppse023	cpfrc_00283	535	52.51	529	Rv0528		NP_215042.1	transmembrane protein

plcppse045	cpfrc_00491	543	73.72	548	Rv3280	*accD5*	NP_217797.1	propionyl-CoA carboxylase beta chain

plcpsec110	cpfrc_00506	362	69.03	359	Rv3264c	*manB*	YP_177951.1	D-alpha-D-mannose-1-phosphate guanylyltransferase MANB

plcpsec111	cpfrc_00508	151	51.45	139	Rv3259		NP_217776.1	hypothetical protein

plcpsec113	cpfrc_00705	487	58.67	495	Rv1018c	*glmU*	NP_215534.1	bifunctional N-acetylglucosamine-1-phosphate uridyltransferase/glucosamine-1-phosphate acetyltransferase

plcpsec115	cpfrc_00945	64	63.33	64	Rv1642	*rpmI*	NP_216158.1	50S ribosomal protein L35

plcppse080	cpfrc_01015	452	57.08	470	Rv0392c	*ndhA*	NP_214906.1	membrane NADH dehydrogenase

plcppse080	cpfrc_01015	452	58.10	463	Rv1854c	*ndh*	NP_216370.1	NADH dehydrogenase

plcpsec041	cpfrc_01074	403	62.96	381	Rv1488		NP_216004.1	hypothetical protein

plcpsec119	cpfrc_01121	504	53.71	457	Rv1407	*fmu*	NP_215923.1	Fmu protein (SUN protein)

plcppse085	cpfrc_01126	417	55.58	418	Rv1391	*dfp*	NP_215907.1	bifunctional phosphopantothenoylcysteine decarboxylase/phosphopantothenate synthase

plcpsec138	cpfrc_01214	79	68.42	82	Rv2708c		NP_217224.1	hypothetical protein

plcpsec122	cpfrc_01267	683	57.76	558	Rv2752c		NP_217268.1	hypothetical protein

plcpsec124	cpfrc_01393	239	57.83	250	Rv2149c	*yfiH*	NP_216665.1	hypothetical protein

plcppse104	cpfrc_01424	412	50.38	429	Rv2195	*qcrA*	NP_216711.1	Rieske iron-sulfur protein QcrA

plcpsec128	cpfrc_01757	313	59.42	322	Rv3579c		NP_218096.1	tRNA/rRNA methyltransferase

plcppse131	cpfrc_01798	480	62.21	491	Rv2443	*dctA*	NP_216959.1	C4-dicarboxylate-transport transmembrane protein DctA

plcppse165	cpfrc_02038	721	52.00	678	Rv0050	*ponA1*	YP_177687.1	bifunctional penicillin-binding protein 1A/1B

plcppse174	cpfrc_02102	393	51.41	406	Rv3915	*cwlM*	YP_178027.1	hydrolase

Related *C. pseudotuberculosis*'s proteins containing at least 50% amino acid identity and 90% alignment size to the *Mtb *H37Rv's proteins.

In this regard, here we only discuss some of these *Mtb*'s genes with experimental evidence. The plcppse174 pan *locus *shows 51% protein identity with Rv3915 (YP_178027.1), a gene named *cwlM *that was the first autolysin gene identified and cloned from *Mtb*. This finding offers a new drug target class that could alter the permeability of the mycobacterium cell wall and enhance the effectiveness of treatments for tuberculosis [26]. Applying principles of *in vivo *expression technology (IVET), it was possible to identify upregulated genes from *Mtb *in an *in vitro *simulation of anaerobic persistence condition. The upregulated genes under hypoxic condition (dissolved oxygen <1%) include Rv0050 (*ponA1*), a penicillin binding protein that has 52% protein identity to the plcppse165 pan *locus *and 90% alignment extension [27]. The plcpsec122 pan *locus *shows ~58% protein identity with Rv2752c (NP_217268.1), a unique bi-functional *Mtb *gene that owns both β-lactamase and RNase activities. Both activities are lost upon deletion of the 100 AA long C-terminal 100 tail, which contains an additional loop when compared to the RNase J of *Bacillus subtilis *[28]. As it can be observed, the plcppse080 pan *locus *appears twice in Table 2, as it is homologous to both NADH dehydrogenase gene copies of *Mtb*, *ndh *(NP_216370.1) and *ndhA *(NP_214906.1), with ~57% protein identity. In *Mtb*, energy generation is mainly performed by type II dehydrogenases *ndh *and *ndhA*, being both, as such, essential genes [29].

The plcpsec113 pan *locus *is homologous to the *glmU *gene (NP_215534.1), holding ~59% protein identity and more than 90% alignment extension. This gene is essential in *Mtb*, being required for optimal bacterial growth, and has been selected as a possible drug target for structural and functional investigation [30]. *GlmU *is a bifunctional acetyltransferase/uridyltransferase that catalyses the formation of UDP-GlcNAc from GlcN-1-P. UDP-GlcNAc is the substrate for two important biosynthetic pathways: lipopolysaccharide and peptidoglycan synthesis. Due to its important roles, *glmU *had its conformational structure solved [30]. The plcpsec113 pan *locus *for *C. pseudotuberculosis *is an interesting putative drug candidate since it is predicted to be secreted, part of the core ISPPE and is able to infer its conformational structure by homology modeling using *Mtb glmU*.

Several genes involved in mannoglycoconjugate biosynthesis have shown to be involved in virulence, due to their central role in biosynthesis of major surface-associated glycoconjugates. Within these genes, the *Mtb *gene *manB *(Rv3264c) is defined as a GDP-mannose pyrophosphorylase (GDPMP) and disruption of its activity leads to decrease of surface-associated mannosylated lipoglycans. For GDPMP, this decrease correspond directly to reduced virulence in both BALB/c mice and cultured human macrophages [31]. The *Mtb manB *gene holds 69% protein identity to the plcpsec110 pan *locus *and more than 90% alignment extension, making plcpsec110 a considerable putative drug target.

Mycolic acids and multimethyl-branched fatty acids are found uniquely in the cell envelope and are essential for survival, virulence and antibiotic resistance of *Mtb*. Acyl-CoA carboxylases (ACCases) commit acyl-CoAs to the biosynthesis of these unique fatty acids. Previous studies indicate that AccD5 is important for cell envelope lipid biosynthesis and its disruption leads to pathogen death [32]. The *Mtb *gene *accD5 *(NP_217797.1) had its structure determined and also shows ~74% protein identity to the plcppse045 pan *locus *in more than 90% alignment extension, making it also a promising candidate for further vaccine candidate evaluations.

Moreover, it was demonstrated that *Mtb *can use heme as an iron source, suggesting that *Mtb *contains a yet-unknown heme acquisition system [33]. We found that the *C. pseudotuberculosis *plcpsec076 pan *locus *holds ~52% protein identity to the *Mtb *gene *hemE *(NP_217194.1) and more than 90% alignment size, therefore also representing an interesting drug target for *C. pseudotuberculosis*.

### Candidates filtering

The here presented results provide a plethora of putative vaccine candidates never seen before for *C. pseudotuberculosis*. However, genes predicted as MEM and CYT account respectively for 18% and 65% of the *in silico *predicted pan genome. Despite the 227 surfaceome and 150 secretome genes here presented, these only represents ~16% of the *C. pseudotuberculosis in silico *predicted pan genome. Most of the genes remain inaccessible for the current *in silico *prediction techniques and it is possible that these neglected genes could also be good candidates against *C. pseudotuberculosis*. These findings raise the need for more elaborated and driven software or prediction schemas capable of uncovering these major genome neglected portions. Using the prediction schema here presented, it was possible to include more than ~2% of non-classic secreted putative proteins that compose putative vaccine candidates. However, this low income amount of vaccine candidates is due to the optional parameter selected in our prediction schema, the non-classic secreted score greater than or equal 0.90. If using the default parameter from the software secretomeP and NClassG+, this income would be increased up to ~6% and the final income of putative vaccine candidates would be ~20%, using a couple of motifs predictors as depicted in Figure 1. The current reverse vaccinology software allows obtaining a number of candidates closer to 20% of the *C. pseudotuberculosis *genome. These considerations raise a question: supposing that novel software for unexplored secretion pathways come into scenario, what is the genome's percentage that could be selected as putative vaccine candidates? Supposing that this percentage reaches 40%, how could the problem of choosing between almost one thousand putative vaccine candidates to be used for the next vaccine production stage for *C. pseudotuberculosis *be solved? This dilemma could be solved by using further software prediction just like those addressing epitopes MHC class I and II allele affinity [34]; however, this could be just a part of the solution. There are chances of solving this dilemma by means of broader vaccine projects, which would take into account particular variables for each target organism in order to minimise research efforts and the number of possible vaccine candidates [35].

### In silico versus non-in silico

It is broadly known that *in silico *genome investigations could give evidence about the genome's function and structure. It is also known that such *in silico *investigations could only be proved or denied by non-*in silico *experiments. Therefore, such reasonable thinking is not a single-hand avenue. Non-*in silico *experiments could be improved by means of more comprehensive or specific approaches with the objective of getting a closer answer to the reality for biological questions. The fact is that *in silico *analyses cannot vary when executed over and over again and no matter how many folds are run. We know that exactly 122 genes will be always predicted as having classical exportation motifs; on the other hand, we cannot expect the same behavior from non-*in silico *analysis. Some real proteins could be or not be found in an *in vitro *or *in vivo *exoproteome result, due to an uncountable number of factors [21]. Therefore, we suggest that the core *C. pseudotuberculosis *ISPPE could be composed of a larger number of predicted genes, but such confirmation could only be affirmed with additional non-*in silico *exoproteome experiments.

## Conclusions

The *in silico *pan-exoproteome prediction methodology applied to the pathogen *C. pseudotuberculosis *helps to raise new insights into putative vaccine candidates against CLA. Additional investigations of the *in vitro *exoproteome of two strains of *C. pseudotuberculosis*, 1002 and C231, showed evidence that the major part of the variant *in vitro *exoproteome is contained in the core ISPPE. A simultaneous curation of the *in silico *predicted core secretome and surfaceome within the five *C. pseudotuberculosis *strains also contributed to homogenize the genome annotations and it was possible to fix the most probable putative methionine proteins. Moreover, putative miss assembled genes, formerly classified as pseudogenes by *in silico *analyses, were also revised. The efforts to create a *C. pseudotuberculosis *ISSPE catalogue proved to be necessary and computationally viable to ensure a uniform set of putative vaccine candidates free of annotation errors.

## Methods

### Genomes

The analyzed *C. pseudotuberculosis *genomes were obtained from the GenBank according to the following accession numbers: EMBL: CP001809 (strain 1002), EMBL: CP001809 (strain C231), EMBL: CP002251 (strain I19), EMBL: CP002924 (strain PAT10) and EMBL: NC_014329 (strain FRC41).

### Prediction schema

Predicted genes from all five *C. pseudotuberculosis *strain genomes were exported as amino acid fasta files using the Artemis software. These fasta files were passed as parameters to SurfG+ 1.0 (Figure 1), and lists of genes predicted as CYT, SEC, PSE and MEM were created by this software. Genes formerly predicted as CYT by SurfG+ were then submitted to the TapP 1.0 predictor; when a Tat motif was found, the putative protein was automatically classified as SEC, otherwise, another prediction round would took place using two other non-classic secretion predictors, SecretomeP 2.0 and NclassG+ 1.0. With a positive prediction from both software and a prediction score greater than or equal to 0.90, the genes were automatically classified as SEC. The SEC and PSE data sets were finally submitted to a reciprocal blastp processing and posterior filtering, giving rise to the fivefold categories according to folds occurring in each strain: 5x, 4x, 3x, 2x and 1x. The results were then manually curated using the ACT software and strain 1002, the first to be sequenced and annotated. The strain 1002 was disposed, in ACT software, in the middle of two pairs of the other two genome strains, facilitating to exhibit differences among all of them.

### SurfG+ 1.0

Sub-cellular localization prediction of *C. pseudotuberculosis *putative proteins was made by *in silico *analysis using the SurfG+ 1.0 software [6]. SurfG+ is a pipeline for protein sub-cellular prediction that incorporates common software, such as SignalP, LipoP and TMHMM to search for motifs. It also creates novel HMMSEARCH profiles to predict cell wall retention signals. SurfG+ starts searching, in the following order for: retention signals, lipoproteins, SEC pathway export motifs and transmembrane motifs. If none of these motifs are found in a protein sequence, it is then characterized as CYT. A novel possible characterization introduced by SurfG+ is its ability to better distinguish between MEM and PSE, by informing an expected cell wall thickness in amino acids. Using the literature or an electronic microscopy it is possible to estimate a reasonable cell wall thickness value for prokaryotic organisms. By means of this last option, *C. pseudotuberculosis *genes were classified into four different sub-cellular locations: CYT, MEM, PSE, or SEC.

### TatP 1.0 Server

Twin-arginine signal peptide motifs were predicted using the on line server hosted by http://www.cbs.dtu.dk/services/TatP/[20]. Only putative proteins formerly classified as CYT by SurfG+ were submitted to the TatP analyses. There were no intersections between SignalP and TatP predictions.

### SecretomeP 2.0 and NClassG+ 1.0

Non-classical secreted putative proteins were predicted using the online server hosted by http://www.cbs.dtu.dk/services/SecretomeP/[21]. NClassG+ [22], a second non-classical secreted protein predictor, was also used; however, the predictions were directly performed contacting the software authors. This double check prediction ensured greater accuracy. Only those genes formerly classified as CYT by SurfG+ and without the twin-arginine signal peptide motifs were submitted to a non-classical secreted analysis. Despite the significant scores of SecretomeP and Nclass+, ranging between 0.5 and 1.0, only those genes with a score greater than or equal to 0.9 were selected, in order to ensure a minimal false positive in future wet lab experiments, the focus of our research group.

### Pan genome

To predict the *C. pseudotuberculosis *pan genome, reciprocal blastp results were used. All the putative proteins predicted as SEC were put apart in a single amino acid fasta file to make a reciprocal blast. A similar file was also created for the proteins predicted as PSE. To avoid homologous mismatches, the blastp results obtained using the PAM70 substitution matrix and the 10^-6 ^e-value were manually filtered. In this regard, the first step was to establish the alignment size and identity percentages of cut-offs, being 89.58 and 50.00%, respectively, for SEC putative proteins, whereas for PSE putative proteins, these cut-offs were 88.16 and 48.80%, respectively. Identity percentages closer to 50% are explained by frame shifts not annotated until this work. All the putative proteins from the five strains (query) with alignment size and identity percentages higher than these cut-offs had no more than one group of blast hits (subject) against the others strains. Moreover, within each of these blast hits groups, there was a blast hit from the query protein against it self as subject. The results were manually curated using the ACT software, from the Artemis package [24], using the strain 1002 as reference strain for the other two strains. This ACT view was composed by strains C231-1002-I19 and FRC41-1002-I19. Each putative protein predicted as SEC and PSE was compared against their other four homologues for correct initial methionine, frame shifts and finally annotating the correct sub-cellular location.

### Revised *in vitro *exoproteome results

In lists 1 and 2 of the annex are both gene *locus *present in the *C. pseudotuberculosis *ISPPE, together with the quantity of homologous genes present in the all five genomes. These results were inserted in a relational database, denominated *C. pseudotuberculosis *Data Base (CpDB) [36], in a specific table called 'exopred'. The list of the *in vitro *exoproteome proteins was also inserted to the CpDB into a table called 'exo' that discriminates the identification of each protein regarding GenBank (protein id), as well as in which strains it is found. To make a relationship between the 'exopred' and 'exo' tables, a third table of the CpDB, called 'gene', which contains all the functional annotation of the genomes of *C. pseudotuberculosis*, was created. The CpDB is the repository of the pan genome of *C. pseudotuberculosis*, harbouring the genomes since their initial genomic prediction, deposited in the GenBank, as well as the annotation corrections for future deposits. For this last purpose, the CpDB stores the identification of each protein according to the GenBank. In this way, it is possible to make a link between the three tables in the form of a clause of JOIN of the SQL: "*... WHERE gene.locus_tag = exopred.locus_tag AND gene.protein_id = exo.protein_id AND exopred.pangenome_coverage = '5x' ..*.". This clause returns the registries of the CpDB whose *locus_tag *in the gene table is equal to the *locus_tag *of the explored table, being this same gene in the protein_id field in the exo table with prediction of belonging to all five genomes. Other conditions can also be included, such as for example, restraining the results to specific genes of a *C. pseudotuberculosis *strain or simultaneously present in the exoproteome of specific strains.

## Competing interests

The authors declare that they have no competing interests.

## Authors' contributions

VA encouraged the research, BMC application, provided references, applied biological knowledge and gave final approval of the version to be published. ARS conducted all the software analyses, manually corrected annotation errors in the five genomes, developed the prediction schema and wrote the paper. EB contributed to the manually curation of all pseudogenes from all bacterial strains. MZT made substantial contributions to the design and interpretation of the manuscript. UP, AG, FD, FS, AC, AP, DB, FF, LG, RR, SA, SS, VCA, AS and AM have given final approval of the version to be published.

## Supplementary Material

Additional file 1 ***C. pseudotuberculosis *genomes**. The five *C. pseudotuberculosis *genomes here checked, as EMBL files. Click here for file

Additional file 2 **Predicted *C. pseudotuberculosis *pan secretome**. List of the 150 genes for 750 *locus_tags *from the five *C. pseudotuberculosis *strains. Click here for file

Additional file 3 **Predicted *C. pseudotuberculosis *pan surfaceome**. List of the 227 genes for 1135 locus_tags from the five *C. pseudotuberculosis *strains. Click here for file
