# Supplementary material for: The Corynebacterium pseudotuberculosis in silico predicted pan-exoproteome
Source: BMC Genomics. 2012 Oct 19;13(Suppl 5):S6. doi: 10.1186/1471-2164-13-S5-S6 (PMC3476999; doi:10.1186/1471-2164-13-S5-S6)
Supplement: Additional file 2 — Predicted C. pseudotuberculosis pan secretome. List of the 150 genes for 750 locus_tags from the five C. pseudotuberculosis strains. [file 1471-2164-13-S5-S6-S2.pdf]

The *Corynebacterium pseudotuberculosis in silico* predicted pan-exoproteome

Anderson Santos[1], Adriana Carneiro[2], Alfonso Gala-García[1], Anne Pinto[1], Debmalya Barh[3], Eudes Barbosa[1], Flávia Figueira[1], Fernanda Dorella[1], Flávia Souza[1], Luis Guimarães[1], Meritxell Turk[1], Rommel Ramos[2], Sintia Almeida[1], Siomar Soares[1], Ulisses Pereira[1], Vinícius Abreu[1], Artur Silva[2], Anderson Miyoshi[1], Vasco Azevedo[1§]

[1]Molecular and Celular Genetics Laboratory, Instituto de Ciências Biológicas, Universidade Federal de Minas Gerais, Belo Horizonte, Minas Gerais, Brazil
[2]DNA Polimorfism Laboratory, Universidade Federal do Pará, Campus do Guamá - Belém, PA, Brazil
[3]Centre for Genomics and Applied Gene Technology, Institute of Integrative Omics and Applied Biotechnology, Nonakuri, Purba Medinipur, West Bengal, India
[§]Corresponding author: vasco@icb.ufmg.br

Additional file 2 – *C. pseudotuberculosis* predicted pan secretome

Set = gene coverage based on homology within five strains (1002, C231, I19, FRC41 and PAT10)

Locus tag	Pan locus	Local subcellular	Predicted by	Set
Cp1002_0024	plcpsec001	SECRETED	SurfG	5x
CpC231_0022	plcpsec001	SECRETED	SurfG	5x
cpfrc_00026	plcpsec001	SECRETED	SurfG	5x
CpI19_0024	plcpsec001	SECRETED	SurfG	5x
CpPAT10_0024	plcpsec001	SECRETED	SurfG	5x
Cp1002_0035	plcpsec002	SECRETED	SurfG	5x
CpC231_0033	plcpsec002	SECRETED	SurfG	5x
cpfrc_00037	plcpsec002	SECRETED	SurfG	5x
CpI19_0035	plcpsec002	SECRETED	SurfG	5x
CpPAT10_0035	plcpsec002	SECRETED	SurfG	5x
Cp1002_0038	plcpsec003	SECRETED	SurfG	5x
CpC231_0036	plcpsec003	SECRETED	SurfG	5x

Locus tag	Pan locus	Local subcellular	Predicted by	Set
cpfrc_00040	plcpsec003	SECRETED	SurfG	5x
CpI19_0038	plcpsec003	SECRETED	SurfG	5x
CpPAT10_0038	plcpsec003	SECRETED	SurfG	5x
Cp1002_0165	plcpsec004	SECRETED	SurfG	5x
CpC231_0168	plcpsec004	SECRETED	SurfG	5x
cpfrc_00167	plcpsec004	SECRETED	SurfG	5x
CpI19_0167	plcpsec004	SECRETED	SurfG	5x
CpPAT10_0168	plcpsec004	SECRETED	SurfG	5x
Cp1002_0185	plcpsec005	SECRETED	SurfG	5x
CpC231_0188	plcpsec005	SECRETED	SurfG	5x
cpfrc_00184	plcpsec005	SECRETED	SurfG	5x
CpI19_0187	plcpsec005	SECRETED	SurfG	5x

Locus tag	Pan locus	Local subcellular	Predicted by	Set
CpPAT10_0188	plcpsec005	SECRETED	SurfG	5x
Cp1002_0193	plcpsec006	SECRETED	SurfG	5x
CpC231_0196	plcpsec006	SECRETED	SurfG	5x
cpfrc_00192	plcpsec006	SECRETED	SurfG	5x
CpI19_0195	plcpsec006	SECRETED	SurfG	5x
CpPAT10_0196	plcpsec006	SECRETED	SurfG	5x
Cp1002_0198	plcpsec007	SECRETED	SurfG	5x
CpC231_0201	plcpsec007	SECRETED	SurfG	5x
cpfrc_00198	plcpsec007	SECRETED	SurfG	5x
CpI19_0200	plcpsec007	SECRETED	SurfG	5x
CpPAT10_0204	plcpsec007	SECRETED	SurfG	5x
Cp1002_0200	plcpsec008	SECRETED	SurfG	5x
CpC231_0203	plcpsec008	SECRETED	SurfG	5x
cpfrc_00200	plcpsec008	SECRETED	SurfG	5x
CpI19_0202	plcpsec008	SECRETED	SurfG	5x
CpPAT10_0206	plcpsec008	SECRETED	SurfG	5x
Cp1002_0208	plcpsec009	SECRETED	SurfG	5x
CpC231_0211	plcpsec009	SECRETED	SurfG	5x
cpfrc_00208	plcpsec009	SECRETED	SurfG	5x
CpI19_0210	plcpsec009	SECRETED	SurfG	5x
CpPAT10_0214	plcpsec009	SECRETED	SurfG	5x
Cp1002_0221	plcpsec010	SECRETED	SurfG	5x
CpC231_0224	plcpsec010	SECRETED	SurfG	5x
cpfrc_00221	plcpsec010	SECRETED	SurfG	5x
CpI19_0223	plcpsec010	SECRETED	SurfG	5x
CpPAT10_0227	plcpsec010	SECRETED	SurfG	5x

Locus tag	Pan locus	Local subcellular	Predicted by	Set
Cp1002_0231	plcpsec011	SECRETED	SurfG	5x
CpC231_0234	plcpsec011	SECRETED	SurfG	5x
cpfrc_00231	plcpsec011	SECRETED	SurfG	5x
CpI19_0233	plcpsec011	SECRETED	SurfG	5x
CpPAT10_0237	plcpsec011	SECRETED	SurfG	5x
Cp1002_0237	plcpsec012	SECRETED	SurfG	5x
CpC231_0240	plcpsec012	SECRETED	SurfG	5x
cpfrc_00237	plcpsec012	SECRETED	SurfG	5x
CpI19_0239	plcpsec012	SECRETED	SurfG	5x
CpPAT10_0243	plcpsec012	SECRETED	SurfG	5x
Cp1002_0249	plcpsec013	SECRETED	SurfG	5x
CpC231_0252	plcpsec013	SECRETED	SurfG	5x
cpfrc_00248	plcpsec013	SECRETED	SurfG	5x
CpI19_0251	plcpsec013	SECRETED	SurfG	5x
CpPAT10_0254	plcpsec013	SECRETED	SurfG	5x
Cp1002_0269	plcpsec014	SECRETED	SurfG	5x
CpC231_0272	plcpsec014	SECRETED	SurfG	5x
cpfrc_00266	plcpsec014	SECRETED	SurfG	5x
CpI19_0271	plcpsec014	SECRETED	SurfG	5x
CpPAT10_0274	plcpsec014	SECRETED	SurfG	5x
Cp1002_0292	plcpsec015	SECRETED	SurfG	5x
CpC231_0295	plcpsec015	SECRETED	SurfG	5x
cpfrc_00289	plcpsec015	SECRETED	SurfG	5x
CpI19_0294	plcpsec015	SECRETED	SurfG	5x
CpPAT10_0297	plcpsec015	SECRETED	SurfG	5x
Cp1002_0368	plcpsec016	SECRETED	SurfG	5x

Locus tag	Pan locus	Local subcellular	Predicted by	Set
CpC231_0371	plcpsec016	SECRETED	SurfG	5x
cpfrc_00366	plcpsec016	SECRETED	SurfG	5x
CpI19_0370	plcpsec016	SECRETED	SurfG	5x
CpPAT10_0372	plcpsec016	SECRETED	SurfG	5x
Cp1002_0376	plcpsec017	SECRETED	SurfG	5x
CpC231_0379	plcpsec017	SECRETED	SurfG	5x
cpfrc_00374	plcpsec017	SECRETED	SurfG	5x
CpI19_0378	plcpsec017	SECRETED	SurfG	5x
CpPAT10_0380	plcpsec017	SECRETED	SurfG	5x
Cp1002_0388	plcpsec018	SECRETED	SurfG	5x
CpC231_0391	plcpsec018	SECRETED	SurfG	5x
cpfrc_00387	plcpsec018	SECRETED	SurfG	5x
CpI19_0390	plcpsec018	SECRETED	SurfG	5x
CpPAT10_0392	plcpsec018	SECRETED	SurfG	5x
Cp1002_0415	plcpsec019	SECRETED	SurfG	5x
CpC231_0418	plcpsec019	SECRETED	SurfG	5x
cpfrc_00415	plcpsec019	SECRETED	SurfG	5x
CpI19_0416	plcpsec019	SECRETED	SurfG	5x
CpPAT10_0419	plcpsec019	SECRETED	SurfG	5x
Cp1002_0535	plcpsec020	SECRETED	SurfG	5x
CpC231_0538	plcpsec020	SECRETED	SurfG	5x
cpfrc_00536	plcpsec020	SECRETED	SurfG	5x
CpI19_0537	plcpsec020	SECRETED	SurfG	5x
CpPAT10_0537	plcpsec020	SECRETED	SurfG	5x
Cp1002_0536	plcpsec021	SECRETED	SurfG	5x
CpC231_0539	plcpsec021	SECRETED	SurfG	5x

Locus tag	Pan locus	Local subcellular	Predicted by	Set
cpfrc_00537	plcpsec021	SECRETED	SurfG	5x
CpI19_0538	plcpsec021	SECRETED	SurfG	5x
CpPAT10_0538	plcpsec021	SECRETED	SurfG	5x
Cp1002_0549	plcpsec022	SECRETED	SurfG	5x
CpC231_0552	plcpsec022	SECRETED	SurfG	5x
cpfrc_00550	plcpsec022	SECRETED	SurfG	5x
CpI19_0551	plcpsec022	SECRETED	SurfG	5x
CpPAT10_0551	plcpsec022	SECRETED	SurfG	5x
Cp1002_0567	plcpsec023	SECRETED	SurfG	5x
CpC231_0569	plcpsec023	SECRETED	SurfG	5x
cpfrc_00567	plcpsec023	SECRETED	SurfG	5x
CpI19_0568	plcpsec023	SECRETED	SurfG	5x
CpPAT10_0568	plcpsec023	SECRETED	SurfG	5x
Cp1002_0573	plcpsec024	SECRETED	SurfG	5x
CpC231_0575	plcpsec024	SECRETED	SurfG	5x
cpfrc_00574	plcpsec024	SECRETED	SurfG	5x
CpI19_0574	plcpsec024	SECRETED	SurfG	5x
CpPAT10_0574	plcpsec024	SECRETED	SurfG	5x
Cp1002_0594	plcpsec025	SECRETED	SurfG	5x
CpC231_0595	plcpsec025	SECRETED	SurfG	5x
cpfrc_00594	plcpsec025	SECRETED	SurfG	5x
CpI19_0594	plcpsec025	SECRETED	SurfG	5x
CpPAT10_0595	plcpsec025	SECRETED	SurfG	5x
Cp1002_0596	plcpsec026	SECRETED	SurfG	5x
CpC231_0597	plcpsec026	SECRETED	SurfG	5x
cpfrc_00597	plcpsec026	SECRETED	SurfG	5x

Locus tag	Pan locus	Local subcellular	Predicted by	Set
CpI19_0596	plcpsec026	SECRETED	SurfG	5x
CpPAT10_0598	plcpsec026	SECRETED	SurfG	5x
Cp1002_0615	plcpsec027	SECRETED	SurfG	5x
CpC231_0615	plcpsec027	SECRETED	SurfG	5x
cpfrc_00617	plcpsec027	SECRETED	SurfG	5x
CpI19_0614	plcpsec027	SECRETED	SurfG	5x
CpPAT10_0616	plcpsec027	SECRETED	SurfG	5x
Cp1002_0666	plcpsec028	SECRETED	SurfG	5x
CpC231_0665	plcpsec028	SECRETED	SurfG	5x
cpfrc_00665	plcpsec028	SECRETED	SurfG	5x
CpI19_0665	plcpsec028	SECRETED	SurfG	5x
CpPAT10_0666	plcpsec028	SECRETED	SurfG	5x
Cp1002_0681	plcpsec029	SECRETED	SurfG	5x
CpC231_0680	plcpsec029	SECRETED	SurfG	5x
cpfrc_00679	plcpsec029	SECRETED	SurfG	5x
CpI19_0680	plcpsec029	SECRETED	SurfG	5x
CpPAT10_0681	plcpsec029	SECRETED	SurfG	5x
Cp1002_0686	plcpsec030	SECRETED	SurfG	5x
CpC231_0685	plcpsec030	SECRETED	SurfG	5x
cpfrc_00684	plcpsec030	SECRETED	SurfG	5x
CpI19_0685	plcpsec030	SECRETED	SurfG	5x
CpPAT10_0686	plcpsec030	SECRETED	SurfG	5x
Cp1002_0720	plcpsec031	SECRETED	SurfG	5x
CpC231_0719	plcpsec031	SECRETED	SurfG	5x
cpfrc_00720	plcpsec031	SECRETED	SurfG	5x
CpI19_0719	plcpsec031	SECRETED	SurfG	5x

Locus tag	Pan locus	Local subcellular	Predicted by	Set
CpPAT10_0718	plcpsec031	SECRETED	SurfG	5x
Cp1002_0763	plcpsec032	SECRETED	SurfG	5x
CpC231_0763	plcpsec032	SECRETED	SurfG	5x
cpfrc_00763	plcpsec032	SECRETED	SurfG	5x
CpI19_0763	plcpsec032	SECRETED	SurfG	5x
CpPAT10_0762	plcpsec032	SECRETED	SurfG	5x
Cp1002_0766	plcpsec033	SECRETED	SurfG	5x
CpC231_0766	plcpsec033	SECRETED	SurfG	5x
cpfrc_00766	plcpsec033	SECRETED	SurfG	5x
CpI19_0766	plcpsec033	SECRETED	SurfG	5x
CpPAT10_0765	plcpsec033	SECRETED	SurfG	5x
Cp1002_0783	plcpsec034	SECRETED	SurfG	5x
CpC231_0783	plcpsec034	SECRETED	SurfG	5x
cpfrc_00783	plcpsec034	SECRETED	SurfG	5x
CpI19_0783	plcpsec034	SECRETED	SurfG	5x
CpPAT10_0782	plcpsec034	SECRETED	SurfG	5x
Cp1002_0824	plcpsec035	SECRETED	SurfG	5x
CpC231_0826	plcpsec035	SECRETED	SurfG	5x
cpfrc_00826	plcpsec035	SECRETED	SurfG	5x
CpI19_0826	plcpsec035	SECRETED	SurfG	5x
CpPAT10_0824	plcpsec035	SECRETED	SurfG	5x
Cp1002_0882	plcpsec036	SECRETED	SurfG	5x
CpC231_0884	plcpsec036	SECRETED	SurfG	5x
cpfrc_00884	plcpsec036	SECRETED	SurfG	5x
CpI19_0885	plcpsec036	SECRETED	SurfG	5x
CpPAT10_0883	plcpsec036	SECRETED	SurfG	5x

Locus tag	Pan locus	Local subcellular	Predicted by	Set
Cp1002_0893	plcpsec037	SECRETED	SurfG	5x
CpC231_0895	plcpsec037	SECRETED	SurfG	5x
cpfrc_00897	plcpsec037	SECRETED	SurfG	5x
CpI19_0896	plcpsec037	SECRETED	SurfG	5x
CpPAT10_0894	plcpsec037	SECRETED	SurfG	5x
Cp1002_0911	plcpsec038	SECRETED	SurfG	5x
CpC231_0915	plcpsec038	SECRETED	SurfG	5x
cpfrc_00916	plcpsec038	SECRETED	SurfG	5x
CpI19_0916	plcpsec038	SECRETED	SurfG	5x
CpPAT10_0912	plcpsec038	SECRETED	SurfG	5x
Cp1002_1000	plcpsec039	SECRETED	SurfG	5x
CpC231_0999	plcpsec039	SECRETED	SurfG	5x
cpfrc_01006	plcpsec039	SECRETED	SurfG	5x
CpI19_1005	plcpsec039	SECRETED	SurfG	5x
CpPAT10_0999	plcpsec039	SECRETED	SurfG	5x
Cp1002_1013	plcpsec040	SECRETED	SurfG	5x
CpC231_1012	plcpsec040	SECRETED	SurfG	5x
cpfrc_01018	plcpsec040	SECRETED	SurfG	5x
CpI19_1018	plcpsec040	SECRETED	SurfG	5x
CpPAT10_1012	plcpsec040	SECRETED	SurfG	5x
Cp1002_1068	plcpsec041	SECRETED	SurfG	5x
CpC231_1066	plcpsec041	SECRETED	SurfG	5x
cpfrc_01074	plcpsec041	SECRETED	SurfG	5x
CpI19_1073	plcpsec041	SECRETED	SurfG	5x
CpPAT10_1067	plcpsec041	SECRETED	SurfG	5x
Cp1002_1144	plcpsec042	SECRETED	SurfG	5x

Locus tag	Pan locus	Local subcellular	Predicted by	Set
CpC231_1143	plcpsec042	SECRETED	SurfG	5x
cpfrc_01148	plcpsec042	SECRETED	SurfG	5x
CpI19_1150	plcpsec042	SECRETED	SurfG	5x
CpPAT10_1143	plcpsec042	SECRETED	SurfG	5x
Cp1002_1234	plcpsec043	SECRETED	SurfG	5x
CpC231_1233	plcpsec043	SECRETED	SurfG	5x
cpfrc_01241	plcpsec043	SECRETED	SurfG	5x
CpI19_1240	plcpsec043	SECRETED	SurfG	5x
CpPAT10_1233	plcpsec043	SECRETED	SurfG	5x
Cp1002_1298	plcpsec044	SECRETED	SurfG	5x
CpC231_1297	plcpsec044	SECRETED	SurfG	5x
cpfrc_01302	plcpsec044	SECRETED	SurfG	5x
CpI19_1303	plcpsec044	SECRETED	SurfG	5x
CpPAT10_1296	plcpsec044	SECRETED	SurfG	5x
Cp1002_1345	plcpsec045	SECRETED	SurfG	5x
CpC231_1344	plcpsec045	SECRETED	SurfG	5x
cpfrc_01351	plcpsec045	SECRETED	SurfG	5x
CpI19_1350	plcpsec045	SECRETED	SurfG	5x
CpPAT10_1344	plcpsec045	SECRETED	SurfG	5x
Cp1002_1378	plcpsec046	SECRETED	SurfG	5x
CpC231_1377	plcpsec046	SECRETED	SurfG	5x
cpfrc_01384	plcpsec046	SECRETED	SurfG	5x
CpI19_1383	plcpsec046	SECRETED	SurfG	5x
CpPAT10_1377	plcpsec046	SECRETED	SurfG	5x
Cp1002_1389	plcpsec047	SECRETED	SurfG	5x
CpC231_1388	plcpsec047	SECRETED	SurfG	5x

**Locus tag**	**Pan locus**	**Local subcellular**	**Predicted by**	**Set**
cpfrc_01395	plcpsec047	SECRETED	SurfG	5x
CpI19_1394	plcpsec047	SECRETED	SurfG	5x
CpPAT10_1388	plcpsec047	SECRETED	SurfG	5x
Cp1002_1416	plcpsec048	SECRETED	SurfG	5x
CpC231_1416	plcpsec048	SECRETED	SurfG	5x
cpfrc_01421	plcpsec048	SECRETED	SurfG	5x
CpI19_1423	plcpsec048	SECRETED	SurfG	5x
CpPAT10_1415	plcpsec048	SECRETED	SurfG	5x
Cp1002_1417	plcpsec049	SECRETED	SurfG	5x
CpC231_1417	plcpsec049	SECRETED	SurfG	5x
cpfrc_01422	plcpsec049	SECRETED	SurfG	5x
CpI19_1424	plcpsec049	SECRETED	SurfG	5x
CpPAT10_1416	plcpsec049	SECRETED	SurfG	5x
Cp1002_1476	plcpsec050	SECRETED	SurfG	5x
CpC231_1478	plcpsec050	SECRETED	SurfG	5x
cpfrc_01485	plcpsec050	SECRETED	SurfG	5x
CpI19_1485	plcpsec050	SECRETED	SurfG	5x
CpPAT10_1478	plcpsec050	SECRETED	SurfG	5x
Cp1002_1506	plcpsec051	SECRETED	SurfG	5x
CpC231_1509	plcpsec051	SECRETED	SurfG	5x
cpfrc_01516	plcpsec051	SECRETED	SurfG	5x
CpI19_1515	plcpsec051	SECRETED	SurfG	5x
CpPAT10_1509	plcpsec051	SECRETED	SurfG	5x
Cp1002_1631	plcpsec052	SECRETED	SurfG	5x
CpC231_1632	plcpsec052	SECRETED	SurfG	5x
cpfrc_01634	plcpsec052	SECRETED	SurfG	5x

**Locus tag**	**Pan locus**	**Local subcellular**	**Predicted by**	**Set**
CpI19_1639	plcpsec052	SECRETED	SurfG	5x
CpPAT10_1632	plcpsec052	SECRETED	SurfG	5x
Cp1002_1745	plcpsec053	SECRETED	SurfG	5x
CpC231_1737	plcpsec053	SECRETED	SurfG	5x
cpfrc_01745	plcpsec053	SECRETED	SurfG	5x
CpI19_1753	plcpsec053	SECRETED	SurfG	5x
CpPAT10_1746	plcpsec053	SECRETED	SurfG	5x
Cp1002_1765	plcpsec054	SECRETED	SurfG	5x
CpC231_1756	plcpsec054	SECRETED	SurfG	5x
cpfrc_01764	plcpsec054	SECRETED	SurfG	5x
CpI19_1773	plcpsec054	SECRETED	SurfG	5x
CpPAT10_1766	plcpsec054	SECRETED	SurfG	5x
Cp1002_1772	plcpsec055	SECRETED	SurfG	5x
CpC231_1762	plcpsec055	SECRETED	SurfG	5x
cpfrc_01770	plcpsec055	SECRETED	SurfG	5x
CpI19_1780	plcpsec055	SECRETED	SurfG	5x
CpPAT10_1772	plcpsec055	SECRETED	SurfG	5x
Cp1002_1802	plcpsec056	SECRETED	SurfG	5x
CpC231_1792	plcpsec056	SECRETED	SurfG	5x
cpfrc_01799	plcpsec056	SECRETED	SurfG	5x
CpI19_1810	plcpsec056	SECRETED	SurfG	5x
CpPAT10_1802	plcpsec056	SECRETED	SurfG	5x
Cp1002_1815	plcpsec057	SECRETED	SurfG	5x
CpC231_1807	plcpsec057	SECRETED	SurfG	5x
cpfrc_01813	plcpsec057	SECRETED	SurfG	5x
CpI19_1825	plcpsec057	SECRETED	SurfG	5x

**Locus tag**	**Pan locus**	**Local subcellular**	**Predicted by**	**Set**
CpPAT10_1817	plcpsec057	SECRETED	SurfG	5x
Cp1002_1820	plcpsec058	SECRETED	SurfG	5x
CpC231_1812	plcpsec058	SECRETED	SurfG	5x
cpfrc_01818	plcpsec058	SECRETED	SurfG	5x
CpI19_1830	plcpsec058	SECRETED	SurfG	5x
CpPAT10_1822	plcpsec058	SECRETED	SurfG	5x
Cp1002_1843	plcpsec059	SECRETED	SurfG	5x
CpC231_1836	plcpsec059	SECRETED	SurfG	5x
cpfrc_01843	plcpsec059	SECRETED	SurfG	5x
CpI19_1854	plcpsec059	SECRETED	SurfG	5x
CpPAT10_1846	plcpsec059	SECRETED	SurfG	5x
Cp1002_1847	plcpsec060	SECRETED	SurfG	5x
CpC231_1840	plcpsec060	SECRETED	SurfG	5x
cpfrc_01847	plcpsec060	SECRETED	SurfG	5x
CpI19_1858	plcpsec060	SECRETED	SurfG	5x
CpPAT10_1850	plcpsec060	SECRETED	SurfG	5x
Cp1002_1852	plcpsec061	SECRETED	SurfG	5x
CpC231_1845	plcpsec061	SECRETED	SurfG	5x
cpfrc_01852	plcpsec061	SECRETED	SurfG	5x
CpI19_1863	plcpsec061	SECRETED	SurfG	5x
CpPAT10_1855	plcpsec061	SECRETED	SurfG	5x
Cp1002_1864	plcpsec062	SECRETED	SurfG	5x
CpC231_1857	plcpsec062	SECRETED	SurfG	5x
cpfrc_01866	plcpsec062	SECRETED	SurfG	5x
CpI19_1875	plcpsec062	SECRETED	SurfG	5x
CpPAT10_1868	plcpsec062	SECRETED	SurfG	5x

**Locus tag**	**Pan locus**	**Local subcellular**	**Predicted by**	**Set**
Cp1002_1893	plcpsec063	SECRETED	SurfG	5x
CpC231_1885	plcpsec063	SECRETED	SurfG	5x
cpfrc_01894	plcpsec063	SECRETED	SurfG	5x
CpI19_1905	plcpsec063	SECRETED	SurfG	5x
CpPAT10_1895	plcpsec063	SECRETED	SurfG	5x
Cp1002_1894	plcpsec064	SECRETED	SurfG	5x
CpC231_1886	plcpsec064	SECRETED	SurfG	5x
cpfrc_01895	plcpsec064	SECRETED	SurfG	5x
CpI19_1906	plcpsec064	SECRETED	SurfG	5x
CpPAT10_1896	plcpsec064	SECRETED	SurfG	5x
Cp1002_1948	plcpsec065	SECRETED	SurfG	5x
CpC231_1942	plcpsec065	SECRETED	SurfG	5x
cpfrc_01951	plcpsec065	SECRETED	SurfG	5x
CpI19_1963	plcpsec065	SECRETED	SurfG	5x
CpPAT10_1955	plcpsec065	SECRETED	SurfG	5x
Cp1002_1955	plcpsec066	SECRETED	SurfG	5x
CpC231_1949	plcpsec066	SECRETED	SurfG	5x
cpfrc_01958	plcpsec066	SECRETED	SurfG	5x
CpI19_1970	plcpsec066	SECRETED	SurfG	5x
CpPAT10_1962	plcpsec066	SECRETED	SurfG	5x
Cp1002_1957	plcpsec067	SECRETED	SurfG	5x
CpC231_1951	plcpsec067	SECRETED	SurfG	5x
cpfrc_01960	plcpsec067	SECRETED	SurfG	5x
CpI19_1972	plcpsec067	SECRETED	SurfG	5x
CpPAT10_1964	plcpsec067	SECRETED	SurfG	5x
Cp1002_1976	plcpsec068	SECRETED	SurfG	5x

Locus tag	Pan locus	Local subcellular	Predicted by	Set
CpC231_1970	plcpsec068	SECRETED	SurfG	5x
cpfrc_01980	plcpsec068	SECRETED	SurfG	5x
CpI19_1991	plcpsec068	SECRETED	SurfG	5x
CpPAT10_1983	plcpsec068	SECRETED	SurfG	5x
Cp1002_2055	plcpsec069	SECRETED	SurfG	5x
CpC231_2049	plcpsec069	SECRETED	SurfG	5x
cpfrc_02056	plcpsec069	SECRETED	SurfG	5x
CpI19_2070	plcpsec069	SECRETED	SurfG	5x
CpPAT10_2059	plcpsec069	SECRETED	SurfG	5x
Cp1002_2064	plcpsec070	SECRETED	SurfG	5x
CpC231_2058	plcpsec070	SECRETED	SurfG	5x
cpfrc_02065	plcpsec070	SECRETED	SurfG	5x
CpI19_2079	plcpsec070	SECRETED	SurfG	5x
CpPAT10_2068	plcpsec070	SECRETED	SurfG	5x
Cp1002_2069	plcpsec071	SECRETED	SurfG	5x
CpC231_2063	plcpsec071	SECRETED	SurfG	5x
cpfrc_02070	plcpsec071	SECRETED	SurfG	5x
CpI19_2084	plcpsec071	SECRETED	SurfG	5x
CpPAT10_2073	plcpsec071	SECRETED	SurfG	5x
Cp1002_2081	plcpsec072	SECRETED	SurfG	5x
CpC231_2074	plcpsec072	SECRETED	SurfG	5x
cpfrc_02081	plcpsec072	SECRETED	SurfG	5x
CpI19_2095	plcpsec072	SECRETED	SurfG	5x
CpPAT10_2084	plcpsec072	SECRETED	SurfG	5x
Cp1002_0126a	plcpsec074	SECRETED	SurfG	5x
CpC231_0129	plcpsec074	SECRETED	SurfG	5x

Locus tag	Pan locus	Local subcellular	Predicted by	Set
cpfrc_00129	plcpsec074	SECRETED	SurfG	5x
CpI19_0129	plcpsec074	SECRETED	SurfG	5x
CpPAT10_0128	plcpsec074	SECRETED	SurfG	5x
Cp1002_0279	plcpsec076	SECRETED	SurfG	5x
CpC231_0282	plcpsec076	SECRETED	SurfG	5x
cpfrc_00276	plcpsec076	SECRETED	SurfG	5x
CpI19_0281	plcpsec076	SECRETED	SurfG	5x
CpPAT10_0284	plcpsec076	SECRETED	SurfG	5x
Cp1002_0699	plcpsec078	SECRETED	SurfG	5x
CpC231_0698	plcpsec078	SECRETED	SurfG	5x
cpfrc_00699	plcpsec078	SECRETED	SurfG	5x
CpI19_0698	plcpsec078	SECRETED	SurfG	5x
CpPAT10_0699	plcpsec078	SECRETED	SurfG	5x
Cp1002_1657	plcpsec082	SECRETED	SurfG	5x
CpC231_1658	plcpsec082	SECRETED	SurfG	5x
cpfrc_01658	plcpsec082	SECRETED	SurfG	5x
CpI19_1666	plcpsec082	SECRETED	SurfG	5x
CpPAT10_1657	plcpsec082	SECRETED	SurfG	5x
Cp1002_1716	plcpsec084	SECRETED	SurfG	5x
CpC231_1708	plcpsec084	SECRETED	SurfG	5x
cpfrc_01715	plcpsec084	SECRETED	SurfG	5x
CpI19_1724	plcpsec084	SECRETED	SurfG	5x
CpPAT10_1716	plcpsec084	SECRETED	SurfG	5x
Cp1002_0027	plcpsec086	SECRETED	SurfG	5x
CpC231_0025	plcpsec086	SECRETED	SurfG	5x
cpfrc_00029	plcpsec086	SECRETED	SurfG	5x

**Locus tag**	**Pan locus**	**Local subcellular**	**Predicted by**	**Set**
CpI19_0027	plcpsec086	SECRETED	SurfG	5x
CpPAT10_0027	plcpsec086	SECRETED	SurfG	5x
Cp1002_0113	plcpsec088	SECRETED	SurfG	5x
CpC231_0116	plcpsec088	SECRETED	SurfG	5x
cpfrc_00116	plcpsec088	SECRETED	SurfG	5x
CpI19_0116	plcpsec088	SECRETED	SurfG	5x
CpPAT10_0115	plcpsec088	SECRETED	SurfG	5x
Cp1002_0593	plcpsec089	SECRETED	SurfG	5x
CpC231_0593	plcpsec089	SECRETED	SurfG	5x
cpfrc_00593	plcpsec089	SECRETED	SurfG	5x
CpI19_0592	plcpsec089	SECRETED	SurfG	5x
CpPAT10_0593	plcpsec089	SECRETED	SurfG	5x
Cp1002_1143	plcpsec090	SECRETED	SurfG	5x
CpC231_1142	plcpsec090	SECRETED	SurfG	5x
cpfrc_01147	plcpsec090	SECRETED	SurfG	5x
CpI19_1149	plcpsec090	SECRETED	SurfG	5x
CpPAT10_1142	plcpsec090	SECRETED	SurfG	5x
Cp1002_1669	plcpsec093	SECRETED	SurfG	5x
CpC231_1669a	plcpsec093	SECRETED	SurfG	5x
cpfrc_01667a	plcpsec093	SECRETED	SurfG	5x
CpI19_1678	plcpsec093	SECRETED	SurfG	5x
CpPAT10_1669	plcpsec093	SECRETED	SurfG	5x
Cp1002_1888	plcpsec094	SECRETED	SurfG	5x
CpC231_1880	plcpsec094	SECRETED	SurfG	5x
cpfrc_01889a	plcpsec094	SECRETED	SurfG	5x
CpI19_1900	plcpsec094	SECRETED	SurfG	5x

**Locus tag**	**Pan locus**	**Local subcellular**	**Predicted by**	**Set**
CpPAT10_1890a	plcpsec094	SECRETED	SurfG	5x
Cp1002_0031	plcpsec073	SECRETED	SurfG	4x
CpC231_0029	plcpsec073	SECRETED	SurfG	4x
cpfrc_00033	plcpsec073	PSE E	SurfG	4x
CpI19_0031	plcpsec073	SECRETED	SurfG	4x
CpPAT10_0031	plcpsec073	SECRETED	SurfG	4x
Cp1002_0182	plcpsec075	SECRETED	SurfG	4x
CpC231_0185	plcpsec075	SECRETED	SurfG	4x
cpfrc_00181	plcpsec075	SECRETED	SurfG	4x
CpI19_0184	plcpsec075	PSEUDOGENE	SurfG	4x
CpPAT10_0185	plcpsec075	SECRETED	SurfG	4x
Cp1002_0387	plcpsec077	SECRETED	SurfG	4x
CpC231_0390	plcpsec077	SECRETED	SurfG	4x
cpfrc_00386	plcpsec077	SECRETED	SurfG	4x
CpI19_0389	plcpsec077	SECRETED	SurfG	4x
CpPAT10_0391	plcpsec077	CYTOPLASMIC	SurfG	4x
Cp1002_0713	plcpsec079	SECRETED	SurfG	4x
CpC231_0712	plcpsec079	SECRETED	SurfG	4x
cpfrc_00713	plcpsec079	PSE C	SurfG	4x
CpI19_0711	plcpsec079	SECRETED	SurfG	4x
CpPAT10_0711	plcpsec079	SECRETED	SurfG	4x
Cp1002_0813	plcpsec080	SECRETED	SurfG	4x
CpC231_0815	plcpsec080	SECRETED	SurfG	4x
cpfrc_00815	plcpsec080	SECRETED	SurfG	4x
CpI19_0815	plcpsec080	SECRETED	SurfG	4x
CpPAT10_0813	plcpsec080	PSE C	SurfG	4x

**Locus tag**	**Pan locus**	**Local subcellular**	**Predicted by**	**Set**
Cp1002_1156	plcpsec081	SECRETED	SurfG	4x
CpC231_1155	plcpsec081	SECRETED	SurfG	4x
cpfrc_01159	plcpsec081	SECRETED	SurfG	4x
CpI19_1162	plcpsec081	SECRETED	SurfG	4x
CpPAT10_1154	plcpsec081	CYTOPLASMIC	SurfG	4x
Cp1002_1688	plcpsec083	SECRETED	SurfG	4x
CpC231	plcpsec083	NOTFOUND	SurfG	4x
cpfrc_01688	plcpsec083	SECRETED	SurfG	4x
CpI19_1696	plcpsec083	SECRETED	SurfG	4x
CpPAT10_1688	plcpsec083	SECRETED	SurfG	4x
Cp1002_1868	plcpsec085	SECRETED	SurfG	5x
CpC231_1862	plcpsec085	SECRETED	SurfG	5x
cpfrc_01871	plcpsec085	SECRETED	SurfG	5x
CpI19_1879	plcpsec085	SECRETED	SurfG	5x
CpPAT10_1873	plcpsec085	SECRETED	SurfG	5x
Cp1002_1811a	plcpsec091	SECRETED	SurfG	4x
CpC231_1803	plcpsec091	SECRETED	SurfG	4x
cpfrc_01809	plcpsec091	SECRETED	SurfG	4x
CpI19_1821	plcpsec091	SECRETED	SurfG	4x
CpPAT10_1813	plcpsec091	CYTOPLASMIC	SurfG	4x
Cp1002_0096	plcpsec087	SECRETED	SurfG	3x
CpC231_0097	plcpsec087	SECRETED	SurfG	3x
cpfrc_00098	plcpsec087	MEMBRANE	SurfG	3x
CpI19_0098	plcpsec087	PSE C	SurfG	3x
CpPAT10_0096	plcpsec087	SECRETED	SurfG	3x
Cp1002_1651	plcpsec092	SECRETED	SurfG	3x

**Locus tag**	**Pan locus**	**Local subcellular**	**Predicted by**	**Set**
CpC231_1652	plcpsec092	SECRETED	SurfG	3x
cpfrc_01652	plcpsec092	CYTOPLASMIC	SurfG	3x
CpI19_1660	plcpsec092	SECRETED	SurfG	3x
CpPAT10_1651	plcpsec092	CYTOPLASMIC	SurfG	3x
Cp1002_1971	plcpsec095	SECRETED	SurfG	3x
CpC231_1965	plcpsec095	SECRETED	SurfG	3x
cpfrc_01975	plcpsec095	SECRETED	SurfG	3x
CpI19_1986	plcpsec095	CYTOPLASMIC	SurfG	3x
CpPAT10_1978	plcpsec095	CYTOPLASMIC	SurfG	3x
Cp1002_0014	plcpsec099	CYTOPLASMIC	SurfG	3x
CpC231_0012	plcpsec099	SECRETED	SurfG	3x
Cpfrc_00012	plcpsec099	CYTOPLASMIC	SurfG	3x
CpI19_0014	plcpsec099	SECRETED	SurfG	3x
CpPAT10_0014	plcpsec099	SECRETED	SurfG	3x
Cp1002_0510	plcpsec101	PSE C	SurfG	3x
CpC231_0514	plcpsec101	SECRETED	SurfG	3x
cpfrc_00513	plcpsec101	SECRETED	SurfG	3x
CpI19_0513	plcpsec101	PSE C	SurfG	3x
CpPAT10_0513	plcpsec101	SECRETED	SurfG	3x
Cp1002_0065	plcpsec096	SECRETED	SurfG	2x
CpC231_0064	plcpsec096	PSE C	SurfG	2x
cpfrc_00067	plcpsec096	SECRETED	SurfG	2x
CpI19_0065	plcpsec096	PSE C	SurfG	2x
CpPAT10_0066	plcpsec096	PSE C	SurfG	2x
Cp1002_1763	plcpsec097	SECRETED	SurfG	2x
CpC231_1754	plcpsec097	SECRETED	SurfG	2x

**Locus tag**	**Pan locus**	**Local subcellular**	**Predicted by**	**Set**
cpfrc_01762	plcpsec097	PSE C	SurfG	2x
CpI19_1771	plcpsec097	PSE C	SurfG	2x
CpPAT10_1764	plcpsec097	PSE E	SurfG	2x
Cp1002_1797	plcpsec098	SECRETED	SurfG	2x
CpC231_1787	plcpsec098	SECRETED	SurfG	2x
cpfrc_01795	plcpsec098	PSE C	SurfG	2x
CpI19_1805	plcpsec098	PSE C	SurfG	2x
CpPAT10_1797	plcpsec098	PSE C	SurfG	2x
Cp1002_0369	plcpsec100	PSEUDOGENE	SurfG	2x
CpC231_0372	plcpsec100	SECRETED	SurfG	2x
cpfrc_00367	plcpsec100	CYTOPLASMIC	SurfG	2x
CpI19_0371	plcpsec100	SECRETED	SurfG	2x
CpPAT10_0373	plcpsec100	PSE C	SurfG	2x
Cp1002_0903	plcpsec102	CYTOPLASMIC	SurfG	2x
CpC231_0905	plcpsec102	SECRETED	SurfG	2x
cpfrc_00907	plcpsec102	SECRETED	SurfG	2x
CpI19_0906	plcpsec102	PSEUDOGENE	SurfG	2x
CpPAT10_0904	plcpsec102	CYTOPLASMIC	SurfG	2x
Cp1002_1310	plcpsec104	PSE C	SurfG	1x
CpC231_1309	plcpsec104	SECRETED	SurfG	1x
cpfrc_01315	plcpsec104	PSE C	SurfG	1x
CpI19_1315	plcpsec104	PSE C	SurfG	1x
CpPAT10_1309	plcpsec104	PSE C	SurfG	1x
Cp1002_0102	plcpsec106	SECRETED	TatP	5x
CpC231_0103	plcpsec106	SECRETED	TatP	5x
cpfrc_00104	plcpsec106	SECRETED	TatP	5x

**Locus tag**	**Pan locus**	**Local subcellular**	**Predicted by**	**Set**
CpI19_0104	plcpsec106	SECRETED	TatP	5x
CpPAT10_0102	plcpsec106	SECRETED	TatP	5x
Cp1002_0172	plcpsec108	SECRETED	TatP	5x
CpC231_0175	plcpsec108	SECRETED	TatP	5x
cpfrc_00174	plcpsec108	SECRETED	TatP	5x
CpI19_0174	plcpsec108	SECRETED	TatP	5x
CpPAT10_0175	plcpsec108	SECRETED	TatP	5x
Cp1002_0502	plcpsec110	SECRETED	TatP	5x
CpC231_0506	plcpsec110	SECRETED	TatP	5x
cpfrc_00506	plcpsec110	SECRETED	TatP	5x
CpI19_0505	plcpsec110	SECRETED	TatP	5x
CpPAT10_0505	plcpsec110	SECRETED	TatP	5x
Cp1002_0505	plcpsec111	SECRETED	TatP	5x
CpC231_0509	plcpsec111	SECRETED	TatP	5x
cpfrc_00508	plcpsec111	SECRETED	TatP	5x
CpI19_0508	plcpsec111	SECRETED	TatP	5x
CpPAT10_0508	plcpsec111	SECRETED	TatP	5x
Cp1002_0664	plcpsec112	SECRETED	TatP	5x
CpC231_0636	plcpsec112	SECRETED	TatP	5x
CpC231_0663	plcpsec112	SECRETED	TatP	5x
CpI19_0663	plcpsec112	SECRETED	TatP	5x
CpPAT10_0664	plcpsec112	SECRETED	TatP	5x
Cp1002_0705	plcpsec113	SECRETED	TatP	5x
CpC231_0704	plcpsec113	SECRETED	TatP	5x
cpfrc_00705	plcpsec113	SECRETED	TatP	5x
CpI19_0704	plcpsec113	SECRETED	TatP	5x

Locus tag	Pan locus	Local subcellular	Predicted by	Set
CpPAT10_0704	plcpsec113	SECRETED	TatP	5x
Cp1002_0940	plcpsec115	SECRETED	TatP	5x
CpC231_0942	plcpsec115	SECRETED	TatP	5x
cpfrc_00945	plcpsec115	SECRETED	TatP	5x
CpI19_0945	plcpsec115	SECRETED	TatP	5x
CpPAT10_0941	plcpsec115	SECRETED	TatP	5x
Cp1002_0972	plcpsec116	SECRETED	TatP	5x
CpC231_0973	plcpsec116	SECRETED	TatP	5x
cpfrc_00978	plcpsec116	SECRETED	TatP	5x
CpI19_0977	plcpsec116	SECRETED	TatP	5x
CpPAT10_0972	plcpsec116	SECRETED	TatP	5x
Cp1002_1051	plcpsec118	SECRETED	TatP	5x
CpC231_1049	plcpsec118	SECRETED	TatP	5x
cpfrc_01056	plcpsec118	SECRETED	TatP	5x
CpI19_1056	plcpsec118	SECRETED	TatP	5x
CpPAT10_1050	plcpsec118	SECRETED	TatP	5x
Cp1002_1117	plcpsec119	SECRETED	TatP	5x
CpC231_1116	plcpsec119	SECRETED	TatP	5x
cpfrc_01121	plcpsec119	SECRETED	TatP	5x
CpI19_1123	plcpsec119	SECRETED	TatP	5x
CpPAT10_1116	plcpsec119	SECRETED	TatP	5x
Cp1002_1137	plcpsec120	SECRETED	TatP	5x
CpC231_1136	plcpsec120	SECRETED	TatP	5x
cpfrc_01141	plcpsec120	SECRETED	TatP	5x
CpI19_1143	plcpsec120	SECRETED	TatP	5x
CpPAT10_1136	plcpsec120	SECRETED	TatP	5x

Locus tag	Pan locus	Local subcellular	Predicted by	Set
Cp1002_1187	plcpsec121	SECRETED	TatP	5x
CpC231_1186	plcpsec121	SECRETED	TatP	5x
cpfrc_01191	plcpsec121	SECRETED	TatP	5x
CpI19_1193	plcpsec121	SECRETED	TatP	5x
CpPAT10_1185	plcpsec121	SECRETED	TatP	5x
Cp1002_1262	plcpsec122	SECRETED	TatP	5x
CpC231_1261	plcpsec122	SECRETED	TatP	5x
cpfrc_01267	plcpsec122	SECRETED	TatP	5x
CpI19_1268	plcpsec122	SECRETED	TatP	5x
CpPAT10_1260	plcpsec122	SECRETED	TatP	5x
Cp1002_1296	plcpsec123	SECRETED	TatP	5x
CpC231_1295	plcpsec123	SECRETED	TatP	5x
cpfrc_01300	plcpsec123	SECRETED	TatP	5x
CpI19_1301	plcpsec123	SECRETED	TatP	5x
CpPAT10_1294	plcpsec123	SECRETED	TatP	5x
Cp1002_1387	plcpsec124	SECRETED	TatP	5x
CpC231_1386	plcpsec124	SECRETED	TatP	5x
cpfrc_01393	plcpsec124	SECRETED	TatP	5x
CpI19_1392	plcpsec124	SECRETED	TatP	5x
CpPAT10_1386	plcpsec124	SECRETED	TatP	5x
Cp1002_1757	plcpsec128	SECRETED	TatP	5x
CpC231_1749	plcpsec128	SECRETED	TatP	5x
cpfrc_01757	plcpsec128	SECRETED	TatP	5x
CpI19_1765	plcpsec128	SECRETED	TatP	5x
CpPAT10_1758	plcpsec128	SECRETED	TatP	5x
Cp1002_1786	plcpsec129	SECRETED	TatP	5x

**Locus tag**	**Pan locus**	**Local subcellular**	**Predicted by**	**Set**
CpC231_1776	plcpsec129	SECRETED	TatP	5x
cpfrc_01784	plcpsec129	SECRETED	TatP	5x
CpI19_1794	plcpsec129	SECRETED	TatP	5x
CpPAT10_1786	plcpsec129	SECRETED	TatP	5x
Cp1002_0132	plcpsec107	CYTOPLASMIC	TatP	4x
CpC231_0135	plcpsec107	SECRETED	TatP	4x
cpfrc_00135	plcpsec107	SECRETED	TatP	4x
CpI19_0135	plcpsec107	SECRETED	TatP	4x
CpPAT10_0137	plcpsec107	SECRETED	TatP	4x
Cp1002_0252	plcpsec109	SECRETED	TatP	4x
CpC231_0255	plcpsec109	SECRETED	TatP	4x
Cpfrc_00251	plcpsec109	CYTOPLASMIC	TatP	4x
CpI19_0254	plcpsec109	SECRETED	TatP	4x
CpPAT10_0257	plcpsec109	SECRETED	TatP	4x
Cp1002_1497	plcpsec125	PSEUDOGENE	TatP	4x
CpC231_1499	plcpsec125	SECRETED	TatP	4x
cpfrc_01506	plcpsec125	SECRETED	TatP	4x
CpI19_1505	plcpsec125	SECRETED	TatP	4x
CpPAT10_1499	plcpsec125	SECRETED	TatP	4x
Cp1002_1004	plcpsec117	SECRETED	TatP	3x
CpC231_1003	plcpsec117	SECRETED	TatP	3x
cpfrc_01010	plcpsec117	SECRETED	TatP	3x
CpI19_1009	plcpsec117	CYTOPLASMIC	TatP	3x
CpPAT10_1003	plcpsec117	CYTOPLASMIC	TatP	3x
Cp1002_1755	plcpsec127	CYTOPLASMIC	TatP	3x
CpC231_1747	plcpsec127	SECRETED	TatP	3x

**Locus tag**	**Pan locus**	**Local subcellular**	**Predicted by**	**Set**
cpfrc_01755	plcpsec127	CYTOPLASMIC	TatP	3x
CpI19_1763	plcpsec127	SECRETED	TatP	3x
CpPAT10_1756	plcpsec127	SECRETED	TatP	3x
Cp1002_0835	plcpsec114	CYTOPLASMIC	TatP	2x
CpC231_0837	plcpsec114	SECRETED	TatP	2x
cpfrc_00837	plcpsec114	SECRETED	TatP	2x
CpI19_0837	plcpsec114	CYTOPLASMIC	TatP	2x
CpPAT10_0835	plcpsec114	CYTOPLASMIC	TatP	2x
Cp1002_0884	plcpsec130	PSEUDOGENE	TatP	2x
CpC231_0886	plcpsec130	PSEUDOGENE	TatP	2x
cpfrc_00886	plcpsec130	PSEUDOGENE	TatP	2x
CpI19_0887	plcpsec130	SECRETED	TatP	2x
CpPAT10_0885	plcpsec130	SECRETED	TatP	2x
Cp1002_1527	plcpsec126	CYTOPLASMIC	TatP	1x
CpC231_1530	plcpsec126	PSEUDOGENE	TatP	1x
cpfrc_01536	plcpsec126	SECRETED	TatP	1x
CpI19_1536	plcpsec126	PSEUDOGENE	TatP	1x
CpPAT10_1530	plcpsec126	PSEUDOGENE	TatP	1x
Cp1002_0048	plcpsec127	SECRETED	SecP	5x
CpC231_0046	plcpsec127	SECRETED	SecP	5x
cpfrc_00050	plcpsec127	SECRETED	SecP	5x
CpI19_0048	plcpsec127	SECRETED	SecP	5x
CpPAT10_0048	plcpsec127	SECRETED	SecP	5x
Cp1002_0058	plcpsec128	SECRETED	SecP	5x
CpC231_0057	plcpsec128	SECRETED	SecP	5x
cpfrc_00060	plcpsec128	SECRETED	SecP	5x

Locus tag	Pan locus	Local subcellular	Predicted by	Set
CpI19_0058	plcpsec128	SECRETED	SecP	5x
CpPAT10_0059	plcpsec128	SECRETED	SecP	5x
Cp1002_0485	plcpsec129	SECRETED	SecP	5x
CpC231_0489	plcpsec129	SECRETED	SecP	5x
cpfrc_00490	plcpsec129	SECRETED	SecP	5x
CpI19_0488	plcpsec129	SECRETED	SecP	5x
CpPAT10_0490	plcpsec129	SECRETED	SecP	5x
Cp1002_0630	plcpsec130	SECRETED	SecP	5x
CpC231_0630	plcpsec130	SECRETED	SecP	5x
cpfrc_00631	plcpsec130	SECRETED	SecP	5x
CpI19_0629	plcpsec130	SECRETED	SecP	5x
CpPAT10_0631	plcpsec130	SECRETED	SecP	5x
Cp1002_0708	plcpsec131	SECRETED	SecP	5x
CpC231_0707	plcpsec131	SECRETED	SecP	5x
Cpfrc_00707a	plcpsec131	SECRETED	SecP	5x
CpI19_0706a	plcpsec131	SECRETED	SecP	5x
CpPAT10_0706a	plcpsec131	SECRETED	SecP	5x
Cp1002_0988	plcpsec132	SECRETED	SurfG	5x
CpC231_0989	plcpsec132	SECRETED	SurfG	5x
cpfrc_00995	plcpsec132	SECRETED	SurfG	5x
CpI19_0993	plcpsec132	SECRETED	SurfG	5x
CpPAT10_0988	plcpsec132	SECRETED	SurfG	5x
Cp1002_0988a	plcpsec133	SECRETED	SecP	5x
CpC231_0989a	plcpsec133	SECRETED	SecP	5x
cpfrc_00996	plcpsec133	SECRETED	SecP	5x
CpI19_0993a	plcpsec133	SECRETED	SecP	5x

Locus tag	Pan locus	Local subcellular	Predicted by	Set
CpPAT10_0988a	plcpsec133	SECRETED	SecP	5x
Cp1002_1034	plcpsec134	SECRETED	SecP	5x
CpC231_1033	plcpsec134	SECRETED	SecP	5x
cpfrc_01038	plcpsec134	SECRETED	SecP	5x
CpI19_1039	plcpsec134	SECRETED	SecP	5x
CpPAT10_1033	plcpsec134	SECRETED	SecP	5x
Cp1002_1082	plcpsec135	SECRETED	SecP	5x
CpC231_1081	plcpsec135	SECRETED	SecP	5x
cpfrc_01086a	plcpsec135	SECRETED	SecP	5x
CpI19_1088	plcpsec135	SECRETED	SecP	5x
CpPAT10_1081	plcpsec135	SECRETED	SecP	5x
Cp1002_1146	plcpsec136	SECRETED	SecP	5x
CpC231_1145	plcpsec136	SECRETED	SecP	5x
cpfrc_01149a	plcpsec136	SECRETED	SecP	5x
CpI19_1152	plcpsec136	SECRETED	SecP	5x
CpPAT10_1144a	plcpsec136	SECRETED	SecP	5x
Cp1002_1159	plcpsec137	SECRETED	SecP	5x
CpC231_1158	plcpsec137	SECRETED	SecP	5x
cpfrc_01162	plcpsec137	SECRETED	SecP	5x
CpI19_1165	plcpsec137	SECRETED	SecP	5x
CpPAT10_1157	plcpsec137	SECRETED	SecP	5x
Cp1002_1208	plcpsec138	SECRETED	SecP	5x
CpC231_1207	plcpsec138	SECRETED	SecP	5x
cpfrc_01214	plcpsec138	SECRETED	SecP	5x
CpI19_1214	plcpsec138	SECRETED	SecP	5x
CpPAT10_1207	plcpsec138	SECRETED	SecP	5x

**Locus tag**	**Pan locus**	**Local subcellular**	**Predicted by**	**Set**
Cp1002_1401	plcpsec139	SECRETED	SecP	5x
CpC231_1400	plcpsec139	SECRETED	SecP	5x
cpfrc_01406a	plcpsec139	SECRETED	SecP	5x
CpI19_1407	plcpsec139	SECRETED	SecP	5x
CpPAT10_1400	plcpsec139	SECRETED	SecP	5x
Cp1002_1462	plcpsec140	SECRETED	SecP	5x
CpC231_1464	plcpsec140	SECRETED	SecP	5x
cpfrc_01472	plcpsec140	SECRETED	SecP	5x
CpI19_1471	plcpsec140	SECRETED	SecP	5x
CpPAT10_1465	plcpsec140	SECRETED	SecP	5x
Cp1002_1483	plcpsec141	SECRETED	SecP	5x
CpC231_1485	plcpsec141	SECRETED	SecP	5x
cpfrc_01492	plcpsec141	SECRETED	SecP	5x
CpI19_1492	plcpsec141	SECRETED	SecP	5x
CpPAT10_1485	plcpsec141	SECRETED	SecP	5x
Cp1002_1667	plcpsec142	SECRETED	SecP	5x
CpC231_1668	plcpsec142	SECRETED	SecP	5x
cpfrc_01666b	plcpsec142	SECRETED	SecP	5x
CpI19_1676	plcpsec142	SECRETED	SecP	5x
CpPAT10_1667	plcpsec142	SECRETED	SecP	5x
Cp1002_1668	plcpsec143	SECRETED	SecP	5x
CpC231_1669	plcpsec143	SECRETED	SecP	5x
cpfrc_01667	plcpsec143	SECRETED	SecP	5x
CpI19_1677	plcpsec143	SECRETED	SecP	5x
CpPAT10_1668	plcpsec143	SECRETED	SecP	5x

**Locus tag**	**Pan locus**	**Local subcellular**	**Predicted by**	**Set**
Cp1002_1721	plcpsec144	SECRETED	SecP	5x
CpC231_1713	plcpsec144	SECRETED	SecP	5x
cpfrc_01720	plcpsec144	SECRETED	SecP	5x
CpI19_1729	plcpsec144	SECRETED	SecP	5x
CpPAT10_1721	plcpsec144	SECRETED	SecP	5x
Cp1002_1751	plcpsec145	SECRETED	SecP	5x
CpC231_1743	plcpsec145	SECRETED	SecP	5x
cpfrc_01751	plcpsec145	SECRETED	SecP	5x
CpI19_1759	plcpsec145	SECRETED	SecP	5x
CpPAT10_1752	plcpsec145	SECRETED	SecP	5x
Cp1002_1923	plcpsec146	SECRETED	SecP	5x
CpC231_1917	plcpsec146	SECRETED	SecP	5x
cpfrc_01928a	plcpsec146	SECRETED	SecP	5x
CpI19_1938	plcpsec146	SECRETED	SecP	5x
CpPAT10_1930	plcpsec146	SECRETED	SecP	5x
Cp1002_2014a	plcpsec148	SECRETED	SecP	5x
CpC231_2009	plcpsec148	SECRETED	SecP	5x
cpfrc_002020a	plcpsec148	SECRETED	SecP	5x
CpI19_2030a	plcpsec148	SECRETED	SecP	5x
CpPAT10_2023	plcpsec148	SECRETED	SecP	5x
Cp1002_1867	plcpsec147	PSE RN	SecP	1x
CpC231_1861	plcpsec147	SECRETED	SecP	1x
cpfrc_01870	plcpsec147	PSE RN	SecP	1x
CpI19_1878	plcpsec147	PSE RN	SecP	1x
CpPAT10_1872	plcpsec147	PSE RN	SecP	1x
