# Supplementary material for: The Corynebacterium pseudotuberculosis in silico predicted pan-exoproteome
Source: BMC Genomics. 2012 Oct 19;13(Suppl 5):S6. doi: 10.1186/1471-2164-13-S5-S6 (PMC3476999; doi:10.1186/1471-2164-13-S5-S6)
Supplement: Additional file 3 — Predicted C. pseudotuberculosis pan surfaceome. List of the 227 genes for 1135 locus_tags from the five C. pseudotuberculosis strains. [file 1471-2164-13-S5-S6-S3.pdf]

The *Corynebacterium pseudotuberculosis in silico* predicted pan-exoproteome

Anderson Santos[1], Adriana Carneiro[2], Alfonso Gala-García[1], Anne Pinto[1], Debmalya Barh[3], Eudes Barbosa[1], Flávia Figueira[1], Fernanda Dorella[1], Flávia Souza[1], Luis Guimarães[1], Meritxell Turk[1], Rommel Ramos[2], Sintia Almeida[1], Siomar Soares[1], Ulisses Pereira[1], Vinícius Abreu[1], Artur Silva[2], Anderson Miyoshi[1], Vasco Azevedo[1§]

[1]Molecular and Celular Genetics Laboratory, Instituto de Ciências Biológicas, Universidade Federal de Minas Gerais, Belo Horizonte, Minas Gerais, Brazil
[2]DNA Polimorfism Laboratory, Universidade Federal do Pará, Campus do Guamá - Belém, PA, Brazil
[3]Centre for Genomics and Applied Gene Technology, Institute of Integrative Omics and Applied Biotechnology, Nonakuri, Purba Medinipur, West Bengal, India
[§]Corresponding author: vasco@icb.ufmg.br

Additional file 3 – *C. pseudotuberculosis* predicted pan surfaceome

Set = gene coverage based on homology within five strains (1002, C231, I19, FRC41 and PAT10)

Locus tag	Pan locus	Set	Local subcellular
Cp1002_0016	plcppse001	5x	PSE E
CpC231_0014	plcppse001	5x	PSE E
cpfrc_00014	plcppse001	5x	PSE E
CpI19_0016	plcppse001	5x	PSE E
CpPAT10_0016	plcppse001	5x	PSE E
Cp1002_0033	plcppse002	5x	PSE C
CpC231_0031	plcppse002	5x	PSE C
cpfrc_00035	plcppse002	5x	PSE C
CpI19_0033	plcppse002	5x	PSE C
CpPAT10_0033	plcppse002	5x	PSE C
Cp1002_0037	plcppse003	5x	PSE N
CpC231_0035	plcppse003	5x	PSE N
cpfrc_00039	plcppse003	5x	PSE N
CpI19_0037	plcppse003	5x	PSE N
CpPAT10_0037	plcppse003	5x	PSE N
Cp1002_0043	plcppse004	5x	PSE E
CpC231_0041	plcppse004	5x	PSE E
cpfrc_00045	plcppse004	5x	PSE E
CpI19_0043	plcppse004	5x	PSE E
CpPAT10_0043	plcppse004	5x	PSE E
Cp1002_0046	plcppse005	5x	PSE N
CpC231_0044	plcppse005	5x	PSE N
cpfrc_00048	plcppse005	5x	PSE N

Locus tag	Pan locus	Set	Local subcellular
CpI19_0046	plcppse005	5x	PSE N
CpPAT10_0046	plcppse005	5x	PSE N
Cp1002_0053	plcppse006	5x	PSE C
CpC231_0052	plcppse006	5x	PSE C
cpfrc_00055	plcppse006	5x	PSE C
CpI19_0053	plcppse006	5x	PSE C
CpPAT10_0054	plcppse006	5x	PSE C
Cp1002_0064	plcppse007	5x	PSE E
CpC231_0063	plcppse007	5x	PSE E
cpfrc_00066	plcppse007	5x	PSE E
CpI19_0064	plcppse007	5x	PSE E
CpPAT10_0065	plcppse007	5x	PSE E
Cp1002_0072	plcppse008	5x	PSE L
CpC231_0072	plcppse008	5x	PSE L
cpfrc_00074	plcppse008	5x	PSE L
CpI19_0073	plcppse008	5x	PSE L
CpPAT10_0073	plcppse008	5x	PSE L
Cp1002_0077	plcppse009	5x	PSE N
CpC231_0077	plcppse009	5x	PSE N
cpfrc_00079	plcppse009	5x	PSE N
CpI19_0078	plcppse009	5x	PSE N
CpPAT10_0078	plcppse009	5x	PSE N
Cp1002_0079	plcppse010	5x	PSE E

Locus tag	Pan locus	Set	Local subcellular
CpC231_0079	plcppse010	5x	PSE E
cpfrc_00081	plcppse010	5x	PSE E
CpI19_0080	plcppse010	5x	PSE E
CpPAT10_0080	plcppse010	5x	PSE E
Cp1002_0099	plcppse011	5x	PSE C
CpC231_0100	plcppse011	5x	PSE C
cpfrc_00101	plcppse011	5x	PSE C
CpI19_0101	plcppse011	5x	PSE C
CpPAT10_0099	plcppse011	5x	PSE C
Cp1002_0125	plcppse012	5x	PSE L
CpC231_0127	plcppse012	5x	PSE L
cpfrc_00128	plcppse012	5x	PSE L
CpI19_0128	plcppse012	5x	PSE L
CpPAT10_0127	plcppse012	5x	PSE L
Cp1002_0134	plcppse013	5x	PSE N
CpC231_0137	plcppse013	5x	PSE N
cpfrc_00136	plcppse013	5x	PSE N
CpI19_0136	plcppse013	5x	PSE N
CpPAT10_0135	plcppse013	5x	PSE N
Cp1002_0164	plcppse014	5x	PSE RN
CpC231_0167	plcppse014	5x	PSE RN
cpfrc_00166	plcppse014	5x	PSE RN
CpI19_0166	plcppse014	5x	PSE RN
CpPAT10_0167	plcppse014	5x	PSE RN
Cp1002_0166	plcppse015	5x	PSE RN
CpC231_0169	plcppse015	5x	PSE RN
cpfrc_00168	plcppse015	5x	PSE RN
CpI19_0168	plcppse015	5x	PSE RN
CpPAT10_0169	plcppse015	5x	PSE RN
Cp1002_0170	plcppse016	5x	PSE RL
CpC231_0173	plcppse016	5x	PSE RN
cpfrc_00172	plcppse016	5x	PSE RN
CpI19_0172	plcppse016	5x	PSE RL
CpPAT10_0173	plcppse016	5x	PSE RL
Cp1002_0192	plcppse017	5x	PSE N
CpC231_0195	plcppse017	5x	PSE N
cpfrc_00191	plcppse017	5x	PSE N
CpI19_0194	plcppse017	5x	PSE N
CpPAT10_0195	plcppse017	5x	PSE N
Cp1002_0210	plcppse018	5x	PSE C

Locus tag	Pan locus	Set	Local subcellular
CpC231_0213	plcppse018	5x	PSE C
cpfrc_00210	plcppse018	5x	PSE C
CpI19_0212	plcppse018	5x	PSE C
CpPAT10_0216	plcppse018	5x	PSE C
Cp1002_0214	plcppse019	5x	PSE N
CpC231_0217	plcppse019	5x	PSE N
cpfrc_00214	plcppse019	5x	PSE N
CpI19_0216	plcppse019	5x	PSE N
CpPAT10_0220	plcppse019	5x	PSE N
Cp1002_0227	plcppse020	5x	PSE C
CpC231_0230	plcppse020	5x	PSE C
cpfrc_00227	plcppse020	5x	PSE C
CpI19_0229	plcppse020	5x	PSE C
CpPAT10_0233	plcppse020	5x	PSE C
Cp1002_0259	plcppse021	5x	PSE C
CpC231_0262	plcppse021	5x	PSE C
cpfrc_00258	plcppse021	5x	PSE C
CpI19_0261	plcppse021	5x	PSE C
CpPAT10_0264	plcppse021	5x	PSE C
Cp1002_0284	plcppse022	5x	PSE E
CpC231_0287	plcppse022	5x	PSE E
cpfrc_00281	plcppse022	5x	PSE E
CpI19_0286	plcppse022	5x	PSE E
CpPAT10_0289	plcppse022	5x	PSE E
Cp1002_0286	plcppse023	5x	PSE L
CpC231_0289	plcppse023	5x	PSE L
cpfrc_00283	plcppse023	5x	PSE L
CpI19_0288	plcppse023	5x	PSE L
CpPAT10_0291	plcppse023	5x	PSE L
Cp1002_0289	plcppse024	5x	PSE E
CpC231_0292	plcppse024	5x	PSE E
cpfrc_00286	plcppse024	5x	PSE E
CpI19_0291	plcppse024	5x	PSE E
CpPAT10_0294	plcppse024	5x	PSE E
Cp1002_0315	plcppse025	5x	PSE RN
CpC231_0319	plcppse025	5x	PSE RN
cpfrc_00313	plcppse025	5x	PSE RN
CpI19_0318	plcppse025	5x	PSE RN
CpPAT10_0320	plcppse025	5x	PSE RN
Cp1002_0317	plcppse026	5x	PSE N

Locus tag	Pan locus	Set	Local subcellular
CpC231_0321	plcppse026	5x	PSE N
cpfrc_00315	plcppse026	5x	PSE N
CpI19_0320	plcppse026	5x	PSE N
CpPAT10_0322	plcppse026	5x	PSE N
Cp1002_0320	plcppse027	5x	PSE N
CpC231_0324	plcppse027	5x	PSE N
cpfrc_00318	plcppse027	5x	PSE N
CpI19_0323	plcppse027	5x	PSE N
CpPAT10_0325	plcppse027	5x	PSE N
Cp1002_0321	plcppse028	5x	PSE L
CpC231_0325	plcppse028	5x	PSE L
cpfrc_00319	plcppse028	5x	PSE L
CpI19_0324	plcppse028	5x	PSE L
CpPAT10_0326	plcppse028	5x	PSE L
Cp1002_0325	plcppse029	5x	PSE C
CpC231_0328	plcppse029	5x	PSE C
cpfrc_00322	plcppse029	5x	PSE C
CpI19_0327	plcppse029	5x	PSE C
CpPAT10_0329	plcppse029	5x	PSE C
Cp1002_0357	plcppse030	5x	PSE E
CpC231_0360	plcppse030	5x	PSE E
cpfrc_00355	plcppse030	5x	PSE E
CpI19_0359	plcppse030	5x	PSE E
CpPAT10_0362	plcppse030	5x	PSE E
Cp1002_0377	plcppse031	5x	PSE E
CpC231_0380	plcppse031	5x	PSE E
cpfrc_00375	plcppse031	5x	PSE E
CpI19_0379	plcppse031	5x	PSE E
CpPAT10_0381	plcppse031	5x	PSE E
Cp1002_0396	plcppse032	5x	PSE C
CpC231_0399	plcppse032	5x	PSE C
cpfrc_00395	plcppse032	5x	PSE C
CpI19_0398	plcppse032	5x	PSE C
CpPAT10_0400	plcppse032	5x	PSE C
Cp1002_0398	plcppse033	5x	PSE N
CpC231_0401	plcppse033	5x	PSE N
cpfrc_00397	plcppse033	5x	PSE N
CpI19_0400	plcppse033	5x	PSE N
CpPAT10_0402	plcppse033	5x	PSE N
Cp1002_0401	plcppse034	5x	PSE C

Locus tag	Pan locus	Set	Local subcellular
CpC231_0404	plcppse034	5x	PSE C
cpfrc_00399	plcppse034	5x	PSE C
CpI19_0402	plcppse034	5x	PSE C
CpPAT10_0404	plcppse034	5x	PSE C
Cp1002_0402	plcppse035	5x	PSE C
CpC231_0405	plcppse035	5x	PSE C
cpfrc_00400	plcppse035	5x	PSE C
CpI19_0403	plcppse035	5x	PSE C
CpPAT10_0405	plcppse035	5x	PSE C
Cp1002_0422	plcppse036	5x	PSE C
CpC231_0425	plcppse036	5x	PSE C
cpfrc_00424	plcppse036	5x	PSE C
CpI19_0423	plcppse036	5x	PSE C
CpPAT10_0426	plcppse036	5x	PSE C
Cp1002_0429	plcppse037	5x	PSE C
CpC231_0432	plcppse037	5x	PSE C
cpfrc_00432	plcppse037	5x	PSE C
CpI19_0430	plcppse037	5x	PSE C
CpPAT10_0434	plcppse037	5x	PSE C
Cp1002_0432	plcppse038	5x	PSE E
CpC231_0435	plcppse038	5x	PSE E
cpfrc_00435	plcppse038	5x	PSE E
CpI19_0433	plcppse038	5x	PSE E
CpPAT10_0437	plcppse038	5x	PSE E
Cp1002_0436	plcppse039	5x	PSE E
CpC231_0439	plcppse039	5x	PSE E
cpfrc_00439	plcppse039	5x	PSE E
CpI19_0437	plcppse039	5x	PSE E
CpPAT10_0441	plcppse039	5x	PSE E
Cp1002_0439	plcppse040	5x	PSE E
CpC231_0443	plcppse040	5x	PSE E
cpfrc_00443	plcppse040	5x	PSE E
CpI19_0441	plcppse040	5x	PSE E
CpPAT10_0444	plcppse040	5x	PSE E
Cp1002_0450	plcppse041	5x	PSE N
CpC231_0454	plcppse041	5x	PSE N
cpfrc_00454	plcppse041	5x	PSE N
CpI19_0452	plcppse041	5x	PSE N
CpPAT10_0455	plcppse041	5x	PSE N
Cp1002_0451	plcppse042	5x	PSE E

Locus tag	Pan locus	Set	Local subcellular
CpC231_0455	plcppse042	5x	PSE E
cpfrc_00455	plcppse042	5x	PSE E
CpI19_0454	plcppse042	5x	PSE E
CpPAT10_0456	plcppse042	5x	PSE E
Cp1002_0463	plcppse043	5x	PSE N
CpC231_0467	plcppse043	5x	PSE N
cpfrc_00468	plcppse043	5x	PSE N
CpI19_0466	plcppse043	5x	PSE N
CpPAT10_0468	plcppse043	5x	PSE N
Cp1002_0480	plcppse044	5x	PSE C
CpC231_0484	plcppse044	5x	PSE C
cpfrc_00485	plcppse044	5x	PSE C
CpI19_0483	plcppse044	5x	PSE C
CpPAT10_0485	plcppse044	5x	PSE C
Cp1002_0486	plcppse045	5x	PSE N
CpC231_0490	plcppse045	5x	PSE N
cpfrc_00491	plcppse045	5x	PSE N
CpI19_0489	plcppse045	5x	PSE N
CpPAT10_0491	plcppse045	5x	PSE N
Cp1002_0497	plcppse046	5x	PSE E
CpC231_0501	plcppse046	5x	PSE E
cpfrc_00502	plcppse046	5x	PSE E
CpI19_0500	plcppse046	5x	PSE E
CpPAT10_0501	plcppse046	5x	PSE E
Cp1002_0499	plcppse047	5x	PSE C
CpC231_0503	plcppse047	5x	PSE C
cpfrc_00504	plcppse047	5x	PSE C
CpI19_0502	plcppse047	5x	PSE C
CpPAT10_0503	plcppse047	5x	PSE C
Cp1002_0516	plcppse048	5x	PSE C
CpC231_0520	plcppse048	5x	PSE C
cpfrc_00519	plcppse048	5x	PSE C
CpI19_0519	plcppse048	5x	PSE C
CpPAT10_0519	plcppse048	5x	PSE C
Cp1002_0539	plcppse049	5x	PSE C
CpC231_0542	plcppse049	5x	PSE C
cpfrc_00540	plcppse049	5x	PSE C
CpI19_0541	plcppse049	5x	PSE C
CpPAT10_0541	plcppse049	5x	PSE C
Cp1002_0550	plcppse050	5x	PSE E

Locus tag	Pan locus	Set	Local subcellular
CpC231_0553	plcppse050	5x	PSE E
cpfrc_00551	plcppse050	5x	PSE E
CpI19_0552	plcppse050	5x	PSE E
CpPAT10_0552	plcppse050	5x	PSE E
Cp1002_0552	plcppse051	5x	PSE C
CpC231_0555	plcppse051	5x	PSE C
cpfrc_00553	plcppse051	5x	PSE C
CpI19_0554	plcppse051	5x	PSE C
CpPAT10_0554	plcppse051	5x	PSE C
Cp1002_0560	plcppse052	5x	PSE C
CpC231_0563	plcppse052	5x	PSE C
cpfrc_00561	plcppse052	5x	PSE C
CpI19_0562	plcppse052	5x	PSE C
CpPAT10_0562	plcppse052	5x	PSE C
Cp1002_0562	plcppse053	5x	PSE C
CpC231_0564	plcppse053	5x	PSE C
cpfrc_00562	plcppse053	5x	PSE C
CpI19_0563	plcppse053	5x	PSE C
CpPAT10_0563	plcppse053	5x	PSE C
Cp1002_0565	plcppse054	5x	PSE N
CpC231_0567	plcppse054	5x	PSE N
cpfrc_00565	plcppse054	5x	PSE N
CpI19_0566	plcppse054	5x	PSE N
CpPAT10_0566	plcppse054	5x	PSE N
Cp1002_0581	plcppse055	5x	PSE L
CpC231_0582	plcppse055	5x	PSE L
cpfrc_00581	plcppse055	5x	PSE L
CpI19_0582	plcppse055	5x	PSE L
CpPAT10_0581	plcppse055	5x	PSE L
Cp1002_0584	plcppse056	5x	PSE E
CpC231_0585	plcppse056	5x	PSE E
cpfrc_00583	plcppse056	5x	PSE E
CpI19_0584	plcppse056	5x	PSE E
CpPAT10_0584	plcppse056	5x	PSE E
Cp1002_0585	plcppse057	5x	PSE E
CpC231_0586	plcppse057	5x	PSE E
cpfrc_00585	plcppse057	5x	PSE E
CpI19_0585	plcppse057	5x	PSE E
CpPAT10_0585	plcppse057	5x	PSE E
Cp1002_0607	plcppse058	5x	PSE E

Locus tag	Pan locus	Set	Local subcellular
CpC231_0608	plcppse058	5x	PSE E
cpfrc_00610	plcppse058	5x	PSE E
CpI19_0607	plcppse058	5x	PSE E
CpPAT10_0609	plcppse058	5x	PSE E
Cp1002_0623	plcppse059	5x	PSE E
CpC231_0623	plcppse059	5x	PSE E
cpfrc_00625	plcppse059	5x	PSE E
CpI19_0622	plcppse059	5x	PSE E
CpPAT10_0624	plcppse059	5x	PSE E
Cp1002_0643	plcppse060	5x	PSE C
CpC231_0642	plcppse060	5x	PSE C
cpfrc_00643	plcppse060	5x	PSE C
CpI19_0642	plcppse060	5x	PSE C
CpPAT10_0643	plcppse060	5x	PSE C
Cp1002_0648	plcppse061	5x	PSE E
CpC231_0647	plcppse061	5x	PSE E
cpfrc_00648	plcppse061	5x	PSE E
CpI19_0647	plcppse061	5x	PSE E
CpPAT10_0648	plcppse061	5x	PSE E
Cp1002_0661	plcppse062	5x	PSE C
CpC231_0660	plcppse062	5x	PSE C
cpfrc_00661	plcppse062	5x	PSE C
CpI19_0660	plcppse062	5x	PSE C
CpPAT10_0661	plcppse062	5x	PSE C
Cp1002_0706	plcppse063	5x	PSE E
CpC231_0705	plcppse063	5x	PSE C
cpfrc_00706	plcppse063	5x	PSE C
CpI19_0705	plcppse063	5x	PSE C
CpPAT10_0705	plcppse063	5x	PSE C
Cp1002_0715	plcppse064	5x	PSE C
CpC231_0714	plcppse064	5x	PSE C
cpfrc_00715	plcppse064	5x	PSE C
CpI19_0714	plcppse064	5x	PSE C
CpPAT10_0713	plcppse064	5x	PSE C
Cp1002_0734	plcppse065	5x	PSE E
CpC231_0733	plcppse065	5x	PSE E
cpfrc_00733	plcppse065	5x	PSE E
CpI19_0733	plcppse065	5x	PSE E
CpPAT10_0731	plcppse065	5x	PSE E
Cp1002_0744	plcppse066	5x	PSE C

Locus tag	Pan locus	Set	Local subcellular
CpC231_0744	plcppse066	5x	PSE C
cpfrc_00744	plcppse066	5x	PSE C
CpI19_0744	plcppse066	5x	PSE C
CpPAT10_0742	plcppse066	5x	PSE C
Cp1002_0749	plcppse067	5x	PSE C
CpC231_0749	plcppse067	5x	PSE C
cpfrc_00748	plcppse067	5x	PSE C
CpI19_0749	plcppse067	5x	PSE C
CpPAT10_0747	plcppse067	5x	PSE C
Cp1002_0759	plcppse068	5x	PSE E
CpC231_0759	plcppse068	5x	PSE E
cpfrc_00759	plcppse068	5x	PSE E
CpI19_0759	plcppse068	5x	PSE E
CpPAT10_0758	plcppse068	5x	PSE E
Cp1002_0797	plcppse069	5x	PSE E
CpC231_0797	plcppse069	5x	PSE E
cpfrc_00797	plcppse069	5x	PSE E
CpI19_0797	plcppse069	5x	PSE E
CpPAT10_0795	plcppse069	5x	PSE E
Cp1002_0849	plcppse070	5x	PSE C
CpC231_0851	plcppse070	5x	PSE C
cpfrc_00851	plcppse070	5x	PSE C
CpI19_0851	plcppse070	5x	PSE C
CpPAT10_0849	plcppse070	5x	PSE C
Cp1002_0876	plcppse071	5x	PSE E
CpC231_0878	plcppse071	5x	PSE E
cpfrc_00878	plcppse071	5x	PSE E
CpI19_0878	plcppse071	5x	PSE E
CpPAT10_0876	plcppse071	5x	PSE E
Cp1002_0923	plcppse072	5x	PSE E
CpC231_0927	plcppse072	5x	PSE E
cpfrc_00928	plcppse072	5x	PSE E
CpI19_0928	plcppse072	5x	PSE E
CpPAT10_0924	plcppse072	5x	PSE E
Cp1002_0930	plcppse073	5x	PSE C
CpC231_0932	plcppse073	5x	PSE C
cpfrc_00934	plcppse073	5x	PSE C
CpI19_0935	plcppse073	5x	PSE C
CpPAT10_0931	plcppse073	5x	PSE C
Cp1002_0942	plcppse074	5x	PSE N

Locus tag	Pan locus	Set	Local subcellular
CpC231_0944	plcppse074	5x	PSE N
cpfrc_00947	plcppse074	5x	PSE N
CpI19_0947	plcppse074	5x	PSE N
CpPAT10_0943	plcppse074	5x	PSE N
Cp1002_0962	plcppse075	5x	PSE N
CpC231_0964	plcppse075	5x	PSE N
cpfrc_00968	plcppse075	5x	PSE N
CpI19_0967	plcppse075	5x	PSE N
CpPAT10_0962	plcppse075	5x	PSE N
Cp1002_0963	plcppse076	5x	PSE C
CpC231_0965	plcppse076	5x	PSE C
cpfrc_00969	plcppse076	5x	PSE C
CpI19_0968	plcppse076	5x	PSE C
CpPAT10_0963	plcppse076	5x	PSE C
Cp1002_0979	plcppse077	5x	PSE C
CpC231_0980	plcppse077	5x	PSE C
cpfrc_00985	plcppse077	5x	PSE C
CpI19_0984	plcppse077	5x	PSE C
CpPAT10_0979	plcppse077	5x	PSE C
Cp1002_0989	plcppse078	5x	PSE RN
CpC231_0990	plcppse078	5x	PSE RN
cpfrc_00997	plcppse078	5x	PSE RN
CpI19_0994	plcppse078	5x	PSE RN
CpPAT10_0989	plcppse078	5x	PSE RN
Cp1002_1002	plcppse079	5x	PSE C
CpC231_1001	plcppse079	5x	PSE C
cpfrc_01008	plcppse079	5x	PSE C
CpI19_1007	plcppse079	5x	PSE C
CpPAT10_1001	plcppse079	5x	PSE C
Cp1002_1009	plcppse080	5x	PSE N
CpC231_1008	plcppse080	5x	PSE N
cpfrc_01015	plcppse080	5x	PSE N
CpI19_1014	plcppse080	5x	PSE N
CpPAT10_1008	plcppse080	5x	PSE N
Cp1002_1017	plcppse081	5x	PSE L
CpC231_1016	plcppse081	5x	PSE L
cpfrc_01021	plcppse081	5x	PSE L
CpI19_1022	plcppse081	5x	PSE L
CpPAT10_1016	plcppse081	5x	PSE L
Cp1002_1052	plcppse082	5x	PSE E

Locus tag	Pan locus	Set	Local subcellular
CpC231_1050	plcppse082	5x	PSE E
cpfrc_01057	plcppse082	5x	PSE E
CpI19_1057	plcppse082	5x	PSE E
CpPAT10_1051	plcppse082	5x	PSE E
Cp1002_1074	plcppse083	5x	PSE N
CpC231_1073	plcppse083	5x	PSE N
cpfrc_01080	plcppse083	5x	PSE N
CpI19_1080	plcppse083	5x	PSE N
CpPAT10_1073	plcppse083	5x	PSE N
Cp1002_1083	plcppse084	5x	PSE N
CpC231_1082	plcppse084	5x	PSE RN
cpfrc_01087	plcppse084	5x	PSE RN
CpI19_1089	plcppse084	5x	PSE RN
CpPAT10_1082	plcppse084	5x	PSE RN
Cp1002_1122	plcppse085	5x	PSE E
CpC231_1121	plcppse085	5x	PSE E
cpfrc_01126	plcppse085	5x	PSE E
CpI19_1128	plcppse085	5x	PSE E
CpPAT10_1121	plcppse085	5x	PSE E
Cp1002_1151	plcppse086	5x	PSE C
CpC231_1150	plcppse086	5x	PSE C
cpfrc_01154	plcppse086	5x	PSE C
CpI19_1157	plcppse086	5x	PSE C
CpPAT10_1149	plcppse086	5x	PSE C
Cp1002_1153	plcppse087	5x	PSE L
CpC231_1152	plcppse087	5x	PSE L
cpfrc_01156	plcppse087	5x	PSE L
CpI19_1159	plcppse087	5x	PSE L
CpPAT10_1151	plcppse087	5x	PSE L
Cp1002_1164	plcppse088	5x	PSE C
CpC231_1163	plcppse088	5x	PSE C
cpfrc_01168	plcppse088	5x	PSE C
CpI19_1170	plcppse088	5x	PSE C
CpPAT10_1162	plcppse088	5x	PSE C
Cp1002_1168	plcppse089	5x	PSE E
CpC231_1167	plcppse089	5x	PSE E
cpfrc_01172	plcppse089	5x	PSE E
CpI19_1174	plcppse089	5x	PSE E
CpPAT10_1166	plcppse089	5x	PSE E
Cp1002_1169	plcppse090	5x	PSE L

Locus tag	Pan locus	Set	Local subcellular
CpC231_1168	plcppse090	5x	PSE L
cpfrc_01173	plcppse090	5x	PSE L
CpI19_1175	plcppse090	5x	PSE L
CpPAT10_1167	plcppse090	5x	PSE L
Cp1002_1170	plcppse091	5x	PSE L
CpC231_1169	plcppse091	5x	PSE L
cpfrc_01174	plcppse091	5x	PSE L
CpI19_1176	plcppse091	5x	PSE L
CpPAT10_1168	plcppse091	5x	PSE L
Cp1002_1173	plcppse092	5x	PSE E
CpC231_1172	plcppse092	5x	PSE E
cpfrc_01177	plcppse092	5x	PSE E
CpI19_1179	plcppse092	5x	PSE E
CpPAT10_1171	plcppse092	5x	PSE E
Cp1002_1188	plcppse093	5x	PSE E
CpC231_1187	plcppse093	5x	PSE E
cpfrc_01192	plcppse093	5x	PSE E
CpI19_1194	plcppse093	5x	PSE E
CpPAT10_1186	plcppse093	5x	PSE E
Cp1002_1189	plcppse094	5x	PSE N
CpC231_1188	plcppse094	5x	PSE N
cpfrc_01193	plcppse094	5x	PSE N
CpI19_1195	plcppse094	5x	PSE N
CpPAT10_1187	plcppse094	5x	PSE N
Cp1002_1230	plcppse095	5x	PSE N
CpC231_1229	plcppse095	5x	PSE N
cpfrc_01238	plcppse095	5x	PSE N
CpI19_1236	plcppse095	5x	PSE N
CpPAT10_1229	plcppse095	5x	PSE N
Cp1002_1260	plcppse096	5x	PSE C
CpC231_1259	plcppse096	5x	PSE C
cpfrc_01265	plcppse096	5x	PSE C
CpI19_1266	plcppse096	5x	PSE C
CpPAT10_1258	plcppse096	5x	PSE C
Cp1002_1281	plcppse097	5x	PSE E
CpC231_1280	plcppse097	5x	PSE E
cpfrc_01285	plcppse097	5x	PSE E
CpI19_1287	plcppse097	5x	PSE E
CpPAT10_1279	plcppse097	5x	PSE E
Cp1002_1319	plcppse098	5x	PSE C

Locus tag	Pan locus	Set	Local subcellular
CpC231_1318	plcppse098	5x	PSE C
cpfrc_01324	plcppse098	5x	PSE C
CpI19_1324	plcppse098	5x	PSE C
CpPAT10_1318	plcppse098	5x	PSE C
Cp1002_1328	plcppse099	5x	PSE C
CpC231_1327	plcppse099	5x	PSE C
cpfrc_01333	plcppse099	5x	PSE C
CpI19_1333	plcppse099	5x	PSE C
CpPAT10_1327	plcppse099	5x	PSE C
Cp1002_1362	plcppse100	5x	PSE E
CpC231_1361	plcppse100	5x	PSE E
cpfrc_01368	plcppse100	5x	PSE E
CpI19_1367	plcppse100	5x	PSE E
CpPAT10_1361	plcppse100	5x	PSE E
Cp1002_1379	plcppse101	5x	PSE E
CpC231_1378	plcppse101	5x	PSE E
cpfrc_01385	plcppse101	5x	PSE E
CpI19_1384	plcppse101	5x	PSE E
CpPAT10_1378	plcppse101	5x	PSE E
Cp1002_1397	plcppse102	5x	PSE C
CpC231_1396	plcppse102	5x	PSE C
cpfrc_01403	plcppse102	5x	PSE C
CpI19_1402	plcppse102	5x	PSE C
CpPAT10_1396	plcppse102	5x	PSE C
Cp1002_1409	plcppse103	5x	PSE N
CpC231_1409	plcppse103	5x	PSE N
cpfrc_01414	plcppse103	5x	PSE N
CpI19_1416	plcppse103	5x	PSE N
CpPAT10_1408	plcppse103	5x	PSE N
Cp1002_1421	plcppse104	5x	PSE C
CpC231_1420	plcppse104	5x	PSE C
cpfrc_01424	plcppse104	5x	PSE C
CpI19_1427	plcppse104	5x	PSE C
CpPAT10_1418	plcppse104	5x	PSE C
Cp1002_1422	plcppse105	5x	PSE L
CpC231_1421	plcppse105	5x	PSE L
cpfrc_01425	plcppse105	5x	PSE L
CpI19_1428	plcppse105	5x	PSE L
CpPAT10_1419	plcppse105	5x	PSE L
Cp1002_1425	plcppse106	5x	PSE C

Locus tag	Pan locus	Set	Local subcellular
CpC231_1424	plcppse106	5x	PSE C
cpfrc_01428	plcppse106	5x	PSE C
CpI19_1431	plcppse106	5x	PSE C
CpPAT10_1422	plcppse106	5x	PSE C
Cp1002_1466	plcppse107	5x	PSE N
CpC231_1468	plcppse107	5x	PSE N
cpfrc_01476	plcppse107	5x	PSE N
CpI19_1475	plcppse107	5x	PSE N
CpPAT10_1469	plcppse107	5x	PSE N
Cp1002_1467	plcppse108	5x	PSE N
CpC231_1469	plcppse108	5x	PSE N
cpfrc_01477	plcppse108	5x	PSE N
CpI19_1476	plcppse108	5x	PSE N
CpPAT10_1470	plcppse108	5x	PSE N
Cp1002_1492	plcppse109	5x	PSE L
CpC231_1494	plcppse109	5x	PSE L
cpfrc_01502	plcppse109	5x	PSE L
CpI19_1501	plcppse109	5x	PSE L
CpPAT10_1494	plcppse109	5x	PSE L
Cp1002_1493	plcppse110	5x	PSE C
CpC231_1495	plcppse110	5x	PSE C
cpfrc_01503	plcppse110	5x	PSE C
CpI19_1502	plcppse110	5x	PSE C
CpPAT10_1495	plcppse110	5x	PSE C
Cp1002_1503	plcppse111	5x	PSE E
CpC231_1506	plcppse111	5x	PSE E
cpfrc_01513	plcppse111	5x	PSE E
CpI19_1512	plcppse111	5x	PSE E
CpPAT10_1506	plcppse111	5x	PSE E
Cp1002_1510	plcppse112	5x	PSE RC
CpC231_1513	plcppse112	5x	PSE RC
cpfrc_01520	plcppse112	5x	PSE RC
CpI19_1519	plcppse112	5x	PSE RC
CpPAT10_1513	plcppse112	5x	PSE RC
Cp1002_1517	plcppse113	5x	PSE L
CpC231_1520	plcppse113	5x	PSE L
cpfrc_01526	plcppse113	5x	PSE L
CpI19_1526	plcppse113	5x	PSE L
CpPAT10_1520	plcppse113	5x	PSE L
Cp1002_1540	plcppse114	5x	PSE E

Locus tag	Pan locus	Set	Local subcellular
CpC231_1543	plcppse114	5x	PSE E
cpfrc_01549	plcppse114	5x	PSE E
CpI19_1548	plcppse114	5x	PSE E
CpPAT10_1543	plcppse114	5x	PSE E
Cp1002_1549	plcppse115	5x	PSE E
CpC231_1551	plcppse115	5x	PSE E
cpfrc_01558	plcppse115	5x	PSE E
CpI19_1556	plcppse115	5x	PSE E
CpPAT10_1551	plcppse115	5x	PSE E
Cp1002_1573	plcppse116	5x	PSE C
CpC231_1575	plcppse116	5x	PSE C
cpfrc_01580	plcppse116	5x	PSE C
CpI19_1580	plcppse116	5x	PSE C
CpPAT10_1575	plcppse116	5x	PSE C
Cp1002_1604	plcppse117	5x	PSE C
CpC231_1606	plcppse117	5x	PSE C
cpfrc_01608	plcppse117	5x	PSE C
CpI19_1612	plcppse117	5x	PSE C
CpPAT10_1605	plcppse117	5x	PSE C
Cp1002_1610	plcppse118	5x	PSE C
CpC231_1611	plcppse118	5x	PSE C
cpfrc_01615	plcppse118	5x	PSE C
CpI19_1617	plcppse118	5x	PSE C
CpPAT10_1610	plcppse118	5x	PSE C
Cp1002_1647	plcppse119	5x	PSE C
CpC231_1648	plcppse119	5x	PSE C
cpfrc_01649	plcppse119	5x	PSE C
CpI19_1656	plcppse119	5x	PSE C
CpPAT10_1648	plcppse119	5x	PSE C
Cp1002_1705	plcppse121	5x	PSE C
CpC231_1697	plcppse121	5x	PSE C
cpfrc_01704	plcppse121	5x	PSE C
CpI19_1713	plcppse121	5x	PSE C
CpPAT10_1705	plcppse121	5x	PSE C
Cp1002_1706	plcppse122	5x	PSE C
CpC231_1698	plcppse122	5x	PSE C
cpfrc_01705	plcppse122	5x	PSE C
CpI19_1714	plcppse122	5x	PSE C
CpPAT10_1706	plcppse122	5x	PSE C
Cp1002_1714	plcppse123	5x	PSE E

Locus tag	Pan locus	Set	Local subcellular
CpC231_1706	plcppse123	5x	PSE E
cpfrc_01713	plcppse123	5x	PSE E
CpI19_1722	plcppse123	5x	PSE E
CpPAT10_1714	plcppse123	5x	PSE E
Cp1002_1741	plcppse124	5x	PSE C
CpC231_1733	plcppse124	5x	PSE C
cpfrc_01740	plcppse124	5x	PSE C
CpI19_1749	plcppse124	5x	PSE C
CpPAT10_1741	plcppse124	5x	PSE C
Cp1002_1749	plcppse125	5x	PSE N
CpC231_1741	plcppse125	5x	PSE N
cpfrc_01749	plcppse125	5x	PSE N
CpI19_1757	plcppse125	5x	PSE N
CpPAT10_1750	plcppse125	5x	PSE N
Cp1002_1753	plcppse126	5x	PSE E
CpC231_1745	plcppse126	5x	PSE E
cpfrc_01753	plcppse126	5x	PSE E
CpI19_1761	plcppse126	5x	PSE E
CpPAT10_1754	plcppse126	5x	PSE E
Cp1002_1764	plcppse127	5x	PSE N
CpC231_1755	plcppse127	5x	PSE N
cpfrc_01763	plcppse127	5x	PSE N
CpI19_1772	plcppse127	5x	PSE N
CpPAT10_1765	plcppse127	5x	PSE N
Cp1002_1768	plcppse128	5x	PSE C
CpC231_1758	plcppse128	5x	PSE C
cpfrc_01766	plcppse128	5x	PSE C
CpI19_1776	plcppse128	5x	PSE C
CpPAT10_1768	plcppse128	5x	PSE C
Cp1002_1780	plcppse129	5x	PSE C
CpC231_1770	plcppse129	5x	PSE C
cpfrc_01778	plcppse129	5x	PSE C
CpI19_1788	plcppse129	5x	PSE C
CpPAT10_1780	plcppse129	5x	PSE C
Cp1002_1794	plcppse130	5x	PSE C
CpC231_1784	plcppse130	5x	PSE C
cpfrc_01792	plcppse130	5x	PSE C
CpI19_1802	plcppse130	5x	PSE C
CpPAT10_1794	plcppse130	5x	PSE C
Cp1002_1800	plcppse131	5x	PSE C

Locus tag	Pan locus	Set	Local subcellular
CpC231_1790	plcppse131	5x	PSE C
cpfrc_01798	plcppse131	5x	PSE C
CpI19_1808	plcppse131	5x	PSE C
CpPAT10_1800	plcppse131	5x	PSE C
Cp1002_1804	plcppse132	5x	PSE N
CpC231_1794	plcppse132	5x	PSE N
cpfrc_01801	plcppse132	5x	PSE N
CpI19_1812	plcppse132	5x	PSE N
CpPAT10_1804	plcppse132	5x	PSE N
Cp1002_1811	plcppse133	5x	PSE RN
CpC231_1802	plcppse133	5x	PSE RN
cpfrc_01808	plcppse133	5x	PSE RN
CpI19_1820	plcppse133	5x	PSE RN
CpPAT10_1812	plcppse133	5x	PSE RN
Cp1002_1825	plcppse134	5x	PSE C
CpC231_1817	plcppse134	5x	PSE C
cpfrc_01823	plcppse134	5x	PSE C
CpI19_1835	plcppse134	5x	PSE C
CpPAT10_1827	plcppse134	5x	PSE C
Cp1002_1845	plcppse135	5x	PSE L
CpC231_1838	plcppse135	5x	PSE L
cpfrc_01845	plcppse135	5x	PSE L
CpI19_1856	plcppse135	5x	PSE L
CpPAT10_1848	plcppse135	5x	PSE L
Cp1002_1846	plcppse136	5x	PSE C
CpC231_1839	plcppse136	5x	PSE C
cpfrc_01846	plcppse136	5x	PSE C
CpI19_1857	plcppse136	5x	PSE C
CpPAT10_1849	plcppse136	5x	PSE C
Cp1002_1869	plcppse137	5x	PSE RL
CpC231_1863	plcppse137	5x	PSE RL
cpfrc_01872	plcppse137	5x	PSE RL
CpI19_1880	plcppse137	5x	PSE RL
CpPAT10_1874	plcppse137	5x	PSE RL
Cp1002_1870	plcppse138	5x	PSE L
CpC231_1864	plcppse138	5x	PSE L
cpfrc_01873	plcppse138	5x	PSE L
CpI19_1881	plcppse138	5x	PSE L
CpPAT10_1875	plcppse138	5x	PSE L
Cp1002_1872	plcppse139	5x	PSE RN

Locus tag	Pan locus	Set	Local subcellular
CpC231_1865	plcppse139	5x	PSE RN
cpfrc_01874	plcppse139	5x	PSE RL
CpI19_1882	plcppse139	5x	PSE RN
CpPAT10_1876	plcppse139	5x	PSE RN
Cp1002_1874	plcppse140	5x	PSE L
CpC231_1867	plcppse140	5x	PSE L
cpfrc_01875	plcppse140	5x	PSE L
CpI19_1884	plcppse140	5x	PSE L
CpPAT10_1877	plcppse140	5x	PSE L
Cp1002_1878	plcppse141	5x	PSE N
CpC231_1871	plcppse141	5x	PSE N
cpfrc_01879	plcppse141	5x	PSE N
CpI19_1888	plcppse141	5x	PSE N
CpPAT10_1881	plcppse141	5x	PSE N
Cp1002_1885	plcppse142	5x	PSE N
CpC231_1877	plcppse142	5x	PSE N
cpfrc_01887	plcppse142	5x	PSE N
CpI19_1897	plcppse142	5x	PSE N
CpPAT10_1888	plcppse142	5x	PSE N
Cp1002_1887	plcppse143	5x	PSE C
CpC231_1879	plcppse143	5x	PSE C
cpfrc_01889	plcppse143	5x	PSE C
CpI19_1899	plcppse143	5x	PSE C
CpPAT10_1890	plcppse143	5x	PSE C
Cp1002_1901	plcppse144	5x	PSE N
CpC231_1893	plcppse144	5x	PSE N
cpfrc_01905	plcppse144	5x	PSE N
CpI19_1914	plcppse144	5x	PSE N
CpPAT10_1906	plcppse144	5x	PSE N
Cp1002_1909	plcppse145	5x	PSE C
CpC231_1903	plcppse145	5x	PSE C
cpfrc_01915	plcppse145	5x	PSE C
CpI19_1924	plcppse145	5x	PSE C
CpPAT10_1916	plcppse145	5x	PSE C
Cp1002_1914	plcppse146	5x	PSE E
CpC231_1908	plcppse146	5x	PSE E
cpfrc_01920	plcppse146	5x	PSE E
CpI19_1929	plcppse146	5x	PSE E
CpPAT10_1921	plcppse146	5x	PSE E
Cp1002_1925	plcppse147	5x	PSE L

Locus tag	Pan locus	Set	Local subcellular
CpC231_1919	plcppse147	5x	PSE L
cpfrc_01930	plcppse147	5x	PSE L
CpI19_1940	plcppse147	5x	PSE L
CpPAT10_1932	plcppse147	5x	PSE L
Cp1002_1926	plcppse148	5x	PSE C
CpC231_1920	plcppse148	5x	PSE C
cpfrc_01931	plcppse148	5x	PSE C
CpI19_1941	plcppse148	5x	PSE C
CpPAT10_1933	plcppse148	5x	PSE C
Cp1002_1933	plcppse149	5x	PSE C
CpC231_1927	plcppse149	5x	PSE C
cpfrc_01937	plcppse149	5x	PSE C
CpI19_1948	plcppse149	5x	PSE C
CpPAT10_1940	plcppse149	5x	PSE C
Cp1002_1936	plcppse150	5x	PSE L
CpC231_1930	plcppse150	5x	PSE L
cpfrc_01939	plcppse150	5x	PSE L
CpI19_1951	plcppse150	5x	PSE L
CpPAT10_1942	plcppse150	5x	PSE L
Cp1002_1938	plcppse151	5x	PSE L
CpC231_1932	plcppse151	5x	PSE L
cpfrc_01941	plcppse151	5x	PSE L
CpI19_1953	plcppse151	5x	PSE L
CpPAT10_1944	plcppse151	5x	PSE L
Cp1002_1939	plcppse152	5x	PSE L
CpC231_1933	plcppse152	5x	PSE L
cpfrc_01942	plcppse152	5x	PSE L
CpI19_1954	plcppse152	5x	PSE L
CpPAT10_1945	plcppse152	5x	PSE L
Cp1002_1945	plcppse153	5x	PSE L
CpC231_1939	plcppse153	5x	PSE L
cpfrc_01948	plcppse153	5x	PSE L
CpI19_1960	plcppse153	5x	PSE L
CpPAT10_1952	plcppse153	5x	PSE L
Cp1002_1954	plcppse154	5x	PSE E
CpC231_1948	plcppse154	5x	PSE E
cpfrc_01957	plcppse154	5x	PSE E
CpI19_1969	plcppse154	5x	PSE E
CpPAT10_1961	plcppse154	5x	PSE E
Cp1002_1958	plcppse155	5x	PSE C

Locus tag	Pan locus	Set	Local subcellular
CpC231_1952	plcppse155	5x	PSE C
cpfrc_01961	plcppse155	5x	PSE C
CpI19_1973	plcppse155	5x	PSE C
CpPAT10_1965	plcppse155	5x	PSE C
Cp1002_1962	plcppse156	5x	PSE N
CpC231_1956	plcppse156	5x	PSE N
cpfrc_01965	plcppse156	5x	PSE N
CpI19_1977	plcppse156	5x	PSE N
CpPAT10_1969	plcppse156	5x	PSE N
Cp1002_1964	plcppse157	5x	PSE RN
CpC231_1958	plcppse157	5x	PSE RN
cpfrc_01967	plcppse157	5x	PSE RN
CpI19_1979	plcppse157	5x	PSE RN
CpPAT10_1971	plcppse157	5x	PSE RN
Cp1002_1965	plcppse158	5x	PSE E
CpC231_1959	plcppse158	5x	PSE E
cpfrc_01968	plcppse158	5x	PSE E
CpI19_1980	plcppse158	5x	PSE E
CpPAT10_1972	plcppse158	5x	PSE E
Cp1002_1970	plcppse159	5x	PSE E
CpC231_1964	plcppse159	5x	PSE E
cpfrc_01973	plcppse159	5x	PSE E
CpI19_1985	plcppse159	5x	PSE E
CpPAT10_1977	plcppse159	5x	PSE E
Cp1002_1982	plcppse160	5x	PSE C
CpC231_1976	plcppse160	5x	PSE C
cpfrc_01986	plcppse160	5x	PSE C
CpI19_1997	plcppse160	5x	PSE C
CpPAT10_1989	plcppse160	5x	PSE C
Cp1002_1983	plcppse161	5x	PSE N
CpC231_1977	plcppse161	5x	PSE N
cpfrc_01987	plcppse161	5x	PSE N
CpI19_1998	plcppse161	5x	PSE N
CpPAT10_1990	plcppse161	5x	PSE N
Cp1002_1984	plcppse162	5x	PSE N
CpC231_1978	plcppse162	5x	PSE N
cpfrc_01988	plcppse162	5x	PSE N
CpI19_1999	plcppse162	5x	PSE N
CpPAT10_1991	plcppse162	5x	PSE N
Cp1002_2005	plcppse163	5x	PSE E

Locus tag	Pan locus	Set	Local subcellular
CpC231_1999	plcppse163	5x	PSE E
cpfrc_02012	plcppse163	5x	PSE E
CpI19_2021	plcppse163	5x	PSE E
CpPAT10_2014	plcppse163	5x	PSE E
Cp1002_2008	plcppse164	5x	PSE C
CpC231_2002	plcppse164	5x	PSE C
cpfrc_02015	plcppse164	5x	PSE C
CpI19_2024	plcppse164	5x	PSE C
CpPAT10_2017	plcppse164	5x	PSE C
Cp1002_2034	plcppse165	5x	PSE C
CpC231_2028	plcppse165	5x	PSE C
cpfrc_02038	plcppse165	5x	PSE C
CpI19_2050	plcppse165	5x	PSE C
CpPAT10_2041	plcppse165	5x	PSE C
Cp1002_2047	plcppse166	5x	PSE L
CpC231_2041	plcppse166	5x	PSE L
cpfrc_02050	plcppse166	5x	PSE L
CpI19_2062	plcppse166	5x	PSE L
CpPAT10_2054	plcppse166	5x	PSE L
Cp1002_2053	plcppse167	5x	PSE L
CpC231_2047	plcppse167	5x	PSE L
cpfrc_02054	plcppse167	5x	PSE L
CpI19_2068	plcppse167	5x	PSE L
CpPAT10_2057	plcppse167	5x	PSE L
Cp1002_2054	plcppse168	5x	PSE R
CpC231_2048	plcppse168	5x	PSE R
cpfrc_02055	plcppse168	5x	PSE R
CpI19_2069	plcppse168	5x	PSE R
CpPAT10_2058	plcppse168	5x	PSE R
Cp1002_2056	plcppse169	5x	PSE C
CpC231_2050	plcppse169	5x	PSE C
cpfrc_02057	plcppse169	5x	PSE C
CpI19_2071	plcppse169	5x	PSE C
CpPAT10_2060	plcppse169	5x	PSE C
Cp1002_2066	plcppse170	5x	PSE C
CpC231_2060	plcppse170	5x	PSE C
cpfrc_02067	plcppse170	5x	PSE C
CpI19_2081	plcppse170	5x	PSE C
CpPAT10_2070	plcppse170	5x	PSE C
Cp1002_2089	plcppse171	5x	PSE C

**Locus tag**	**Pan locus**	**Set**	**Local subcellular**
CpC231_2082	plcppse171	5x	PSE E
cpfrc_02089	plcppse171	5x	PSE E
CpI19_2103	plcppse171	5x	PSE E
CpPAT10_2092	plcppse171	5x	PSE E
Cp1002_2097	plcppse172	5x	PSE N
CpC231_2090	plcppse172	5x	PSE N
cpfrc_02097	plcppse172	5x	PSE N
CpI19_2111	plcppse172	5x	PSE N
CpPAT10_2100	plcppse172	5x	PSE N
Cp1002_2098	plcppse173	5x	PSE C
CpC231_2091	plcppse173	5x	PSE C
cpfrc_02098	plcppse173	5x	PSE C
CpI19_2112	plcppse173	5x	PSE C
CpPAT10_2101	plcppse173	5x	PSE C
Cp1002_2102	plcppse174	5x	PSE R
CpC231_2095	plcppse174	5x	PSE R
cpfrc_02102	plcppse174	5x	PSE R
CpI19_2116	plcppse174	5x	PSE R
CpPAT10_2105	plcppse174	5x	PSE R
Cp1002_0050a	plcppse176	5x	PSE C
CpC231_0049	plcppse176	5x	PSE C
cpfrc_00052	plcppse176	5x	PSE C
CpI19_0051a	plcppse176	5x	PSE C
CpPAT10_0051	plcppse176	5x	PSE C
Cp1002_1829a	plcppse187	5x	PSE E
CpC231_1822	plcppse187	5x	PSE E
cpfrc_01828	plcppse187	5x	PSE E
CpI19_1840	plcppse187	5x	PSE E
CpPAT10_1832	plcppse187	5x	PSE E
Cp1002_1880	plcppse195	5x	PSE L
CpC231_1873	plcppse195	5x	PSE L
cpfrc_01881	plcppse195	5x	PSE N
CpI19_1890	plcppse195	5x	PSE L
CpPAT10_1883	plcppse195	5x	PSE L
Cp1002_2058	plcppse196	5x	PSE RN
CpC231_2052	plcppse196	5x	PSE RN
cpfrc_02059	plcppse196	5x	PSE RN
CpI19_2073	plcppse196	5x	PSE RN
CpPAT10_2062	plcppse196	5x	PSE RN
Cp1002_0300	plcppse198	5x	PSE E

**Locus tag**	**Pan locus**	**Set**	**Local subcellular**
CpC231_0304	plcppse198	5x	PSE E
cpfrc_00297	plcppse198	5x	PSE E
CpI19_0302	plcppse198	5x	PSE E
CpPAT10_0305	plcppse198	5x	PSE E
Cp1002_0438a	plcppse200	5x	PSE C
CpC231_0441a	plcppse200	5x	PSE C
cpfrc_00442	plcppse200	5x	PSE C
CpI19_0440	plcppse200	5x	PSE C
CpPAT10_0443a	plcppse200	5x	PSE C
Cp1002_0981	plcppse201	5x	PSE E
CpC231_0982	plcppse201	5x	PSE E
cpfrc_00987	plcppse201	5x	PSE E
CpI19_0986	plcppse201	5x	PSE E
CpPAT10_0981	plcppse201	5x	PSE E
Cp1002_1398	plcppse202	5x	PSE C
CpC231_1397	plcppse202	5x	PSE C
cpfrc_01404	plcppse202	5x	PSE C
CpI19_1403	plcppse202	5x	PSE C
CpPAT10_1397	plcppse202	5x	PSE C
Cp1002_0887	plcppse207	5x	PSE C
CpC231_0888	plcppse207	5x	PSE C
cpfrc_00888a	plcppse207	5x	PSE C
CpI19_0890	plcppse207	5x	PSE C
CpPAT10_0888	plcppse207	5x	PSE C
Cp1002_0902	plcppse221	5x	PSE C
CpC231_0904	plcppse221	5x	PSE C
cpfrc_00906	plcppse221	5x	PSE C
CpI19_0905	plcppse221	5x	PSE C
CpPAT10_0903	plcppse221	5x	PSE C
Cp1002_1637	plcppse222	5x	PSE C
CpC231_1638	plcppse222	5x	PSE E
cpfrc_01639	plcppse222	5x	PSE C
CpI19_1646	plcppse222	5x	PSE E
CpPAT10_1638	plcppse222	5x	PSE E
Cp1002_1687	plcppse120	4x	PSE RL
CpC231_1686	plcppse120	4x	PSE C
cpfrc_01685	plcppse120	4x	PSEUDOGENE
CpI19_1695	plcppse120	4x	PSE RL
CpPAT10_1687	plcppse120	4x	PSE RL
Cp1002_0019	plcppse175	4x	PSE C

Locus tag	Pan locus	Set	Local subcellular
CpC231_0017	plcppse175	4x	PSE C
cpfrc_00017	plcppse175	4x	PSE C
CpI19_0019	plcppse175	4x	MEMBRANE
CpPAT10_0019	plcppse175	4x	PSE C
Cp1002_0052	plcppse177	4x	PSE E
CpC231_0051	plcppse177	4x	PSE E
cpfrc_00054	plcppse177	4x	PSE E
CpI19_0052	plcppse177	4x	MEMBRANE
CpPAT10_0053	plcppse177	4x	PSE E
Cp1002_0098	plcppse178	4x	PSE E
CpC231_0099	plcppse178	4x	PSE E
cpfrc_00100	plcppse178	4x	PSE E
CpI19_0100	plcppse178	4x	PSE E
CpPAT10_0098	plcppse178	4x	CYTOPLASMIC
Cp1002_0430	plcppse179	4x	PSE C
CpC231_0433	plcppse179	4x	PSE C
cpfrc_00433	plcppse179	4x	PSE C
CpI19_0431	plcppse179	4x	PSE C
CpPAT10_0435	plcppse179	4x	MEMBRANE
Cp1002_0454	plcppse180	4x	PSE N
CpC231_0458	plcppse180	4x	PSE N
cpfrc_00458	plcppse180	4x	PSE N
CpI19_0457	plcppse180	4x	PSE N
CpPAT10_0459	plcppse180	4x	PSEUDOGENE
Cp1002_0517	plcppse181	4x	PSE E
CpC231_0521	plcppse181	4x	CYTOPLASMIC
cpfrc_00520	plcppse181	4x	PSE E
CpI19_0520	plcppse181	4x	PSE E
CpPAT10_0520	plcppse181	4x	PSE E
Cp1002_0799	plcppse182	4x	PSE C
CpC231_0799	plcppse182	4x	PSE C
cpfrc_00799	plcppse182	4x	PSE C
CpI19_0799	plcppse182	4x	PSE C
CpPAT10_0797	plcppse182	4x	PSEUDOGENE
Cp1002_0810	plcppse183	4x	PSE C
CpC231_0812	plcppse183	4x	PSE C
cpfrc_00812	plcppse183	4x	PSE C
CpI19_0812	plcppse183	4x	PSE C
CpPAT10_0810	plcppse183	4x	MEMBRANE
Cp1002_1018	plcppse184	4x	PSE C

Locus tag	Pan locus	Set	Local subcellular
CpC231_1017	plcppse184	4x	PSE C
cpfrc_01022	plcppse184	4x	PSE C
CpI19_1023	plcppse184	4x	MEMBRANE
CpPAT10_1017	plcppse184	4x	PSE C
Cp1002_1310	plcppse185	4x	PSE C
CpC231_1309	plcppse185	4x	SECRETED
cpfrc_01315	plcppse185	4x	PSE C
CpI19_1315	plcppse185	4x	PSE C
CpPAT10_1309	plcppse185	4x	PSE C
ABSENT	plcppse186	4x	ABSENT
Cp1002_1693	plcppse186	4x	PSE L
cpfrc_01693	plcppse186	4x	PSE L
CpI19_1701	plcppse186	4x	PSE L
CpPAT10_1693	plcppse186	4x	PSE L
Cp1002_1851	plcppse188	4x	PSE C
CpC231_1844	plcppse188	4x	PSE C
cpfrc_01851	plcppse188	4x	PSE C
CpI19_1862	plcppse188	4x	PSEUDOGENE
CpPAT10_1854	plcppse188	4x	PSE C
Cp1002_1867	plcppse189	4x	PSE RN
CpC231_1861	plcppse189	4x	SECRETED
cpfrc_01870	plcppse189	4x	PSE RN
CpI19_1878	plcppse189	4x	PSE RN
CpPAT10_1872	plcppse189	4x	PSE RN
Cp1002_1910	plcppse190	4x	PSE C
CpC231_1904	plcppse190	4x	PSE C
cpfrc_01916	plcppse190	4x	PSE C
CpI19_1925	plcppse190	4x	MEMBRANE
CpPAT10_1917	plcppse190	4x	PSE C
Cp1002_1953	plcppse191	4x	PSE C
CpC231_1947	plcppse191	4x	PSE C
cpfrc_01956	plcppse191	4x	PSE C
CpI19_1968	plcppse191	4x	PSE C
CpPAT10_1960	plcppse191	4x	PSEUDOGENE
Cp1002_0316	plcppse204	4x	PSE E
CpC231_0320	plcppse204	4x	PSE E
cpfrc_00314	plcppse204	4x	CYTOPLASMIC
CpI19_0319	plcppse204	4x	PSE E
CpPAT10_0321	plcppse204	4x	PSE E
Cp1002_1684	plcppse211	4x	PSE C

Locus tag	Pan locus	Set	Local subcellular
CpC231_1684	plcppse211	4x	PSE C
cpfrc_01681	plcppse211	4x	PSE C
CpI19_1693	plcppse211	4x	CYTOPLASMIC
CpPAT10_1684	plcppse211	4x	PSE C
Cp1002_1905	plcppse215	4x	PSE N
CpC231_1897	plcppse215	4x	PSE N
cpfrc_01910	plcppse215	4x	PSE N
CpI19_1918	plcppse215	4x	MEMBRANE
CpPAT10_1910	plcppse215	4x	PSE N
Cp1002_2040	plcppse217	4x	PSE C
CpC231_2034	plcppse217	4x	PSE C
cpfrc_02044	plcppse217	4x	CYTOPLASMIC
CpI19_2056	plcppse217	4x	PSE C
CpPAT10_2047	plcppse217	4x	PSE C
Cp1002_1684a	plcppse228	4x	PSE C
CpC231_1684a	plcppse228	4x	PSE C
cpfrc_01682	plcppse228	4x	PSE C
CpI19_1693a	plcppse228	4x	MEMBRANE
CpPAT10_1685	plcppse228	4x	PSE C
Cp1002_0662	plcppse192	3x	CYTOPLASMIC
CpC231_0661	plcppse192	3x	PSE C
cpfrc_00662	plcppse192	3x	PSE C
CpI19_0661	plcppse192	3x	PSE C
CpPAT10_0662	plcppse192	3x	CYTOPLASMIC
Cp1002_1763	plcppse193	3x	SECRETED
CpC231_1754	plcppse193	3x	SECRETED
cpfrc_01762	plcppse193	3x	PSE C
CpI19_1771	plcppse193	3x	PSE C
CpPAT10_1764	plcppse193	3x	PSE E
Cp1002_1797	plcppse194	3x	SECRETED
CpC231_1787	plcppse194	3x	SECRETED
cpfrc_01795	plcppse194	3x	PSE C
CpI19_1805	plcppse194	3x	PSE C
CpPAT10_1797	plcppse194	3x	PSE C
Cp1002_0219	plcppse197	3x	MEMBRANE
CpC231_0222	plcppse197	3x	PSE N
cpfrc_00219	plcppse197	3x	PSE N
CpI19_0221	plcppse197	3x	MEMBRANE
CpPAT10_0225	plcppse197	3x	PSE N
Cp1002_0065	plcppse218	3x	SECRETED

Locus tag	Pan locus	Set	Local subcellular
CpC231_0064	plcppse218	3x	PSE C
cpfrc_00067	plcppse218	3x	SECRETED
CpI19_0065	plcppse218	3x	PSE C
CpPAT10_0066	plcppse218	3x	PSE C
Cp1002_1838	plcppse223	3x	PSEUDOGENE
CpC231_1831	plcppse223	3x	PSE C
cpfrc_01837	plcppse223	3x	PSEUDOGENE
CpI19_1849	plcppse223	3x	PSE C
CpPAT10_1841	plcppse223	3x	PSE C
Cp1002_0437	plcppse199	2x	PSE E
CpC231_0440	plcppse199	2x	CYTOPLASMIC
cpfrc_00440	plcppse199	2x	PSE E
CpI19_0438	plcppse199	2x	CYTOPLASMIC
CpPAT10_0442	plcppse199	2x	CYTOPLASMIC
Cp1002_1900	plcppse203	2x	PSE RN
CpC231_1892	plcppse203	2x	PSEUDOGENE
cpfrc_01904	plcppse203	2x	PSE RN
CpI19_1913	plcppse203	2x	PSEUDOGENE
CpPAT10_1905	plcppse203	2x	PSEUDOGENE
Cp1002_0510	plcppse205	2x	PSE C
CpC231_0514	plcppse205	2x	SECRETED
cpfrc_00513	plcppse205	2x	SECRETED
CpI19_0513	plcppse205	2x	PSE C
CpPAT10_0513	plcppse205	2x	SECRETED
Cp1002_1859	plcppse212	2x	PSE RN
CpC231_1852	plcppse212	2x	PSE RN
cpfrc_01860	plcppse212	2x	PSEUDOGENE
CpI19_1870	plcppse212	2x	PSEUDOGENE
CpPAT10_1863	plcppse212	2x	PSEUDOGENE
Cp1002_1904	plcppse214	2x	PSE C
CpC231_1896	plcppse214	2x	PSE C
cpfrc_01908	plcppse214	2x	PSEUDOGENE
CpI19_1917	plcppse214	2x	PSEUDOGENE
CpPAT10_1909	plcppse214	2x	PSEUDOGENE
Cp1002_0559	plcppse219	2x	MEMBRANE
CpC231_0562	plcppse219	2x	PSE N
cpfrc_00560	plcppse219	2x	MEMBRANE
CpI19_0561	plcppse219	2x	MEMBRANE
CpPAT10_0561	plcppse219	2x	PSE N
Cp1002_0624	plcppse220	2x	MEMBRANE

**Locus tag**	**Pan locus**	**Set**	**Local subcellular**
CpC231_0624	plcppse220	2x	PSE N
cpfrc_00626	plcppse220	2x	MEMBRANE
CpI19_0623	plcppse220	2x	MEMBRANE
CpPAT10_0625	plcppse220	2x	PSE N
Cp1002_1204	plcppse206	1x	CYTOPLASMIC
CpC231_1203	plcppse206	1x	CYTOPLASMIC
cpfrc_01210	plcppse206	1x	PSE C
CpI19_1210	plcppse206	1x	CYTOPLASMIC
CpPAT10_1203	plcppse206	1x	CYTOPLASMIC
Cp1002_1562	plcppse208	1x	MEMBRANE
CpC231_1564	plcppse208	1x	MEMBRANE
cpfrc_01569	plcppse208	1x	PSE C
CpI19_1569	plcppse208	1x	MEMBRANE
CpPAT10_1564	plcppse208	1x	MEMBRANE
Cp1002_2065	plcppse209	1x	MEMBRANE
CpC231_2059	plcppse209	1x	MEMBRANE
cpfrc_02066	plcppse209	1x	PSE N
CpI19_2080	plcppse209	1x	MEMBRANE
CpPAT10_2069	plcppse209	1x	MEMBRANE
Cp1002_1058	plcppse210	1x	PSE N
CpC231_1056	plcppse210	1x	CYTOPLASMIC
cpfrc_01063	plcppse210	1x	CYTOPLASMIC
CpI19_1063	plcppse210	1x	CYTOPLASMIC
CpPAT10_1057	plcppse210	1x	CYTOPLASMIC
Cp1002_1883	plcppse213	1x	PSE C

**Locus tag**	**Pan locus**	**Set**	**Local subcellular**
CpC231_1875	plcppse213	1x	MEMBRANE
cpfrc_01883	plcppse213	1x	MEMBRANE
CpI19_1893	plcppse213	1x	MEMBRANE
CpPAT10_1885	plcppse213	1x	MEMBRANE
Cp1002_0096	plcppse224	1x	SECRETED
CpC231_0097	plcppse224	1x	SECRETED
cpfrc_00098	plcppse224	1x	MEMBRANE
CpI19_0098	plcppse224	1x	PSE C
CpPAT10_0096	plcppse224	1x	SECRETED
Cp1002_0369	plcppse226	1x	PSEUDOGENE
CpC231_0372	plcppse226	1x	SECRETED
cpfrc_00367	plcppse226	1x	CYTOPLASMIC
CpI19_0371	plcppse226	1x	SECRETED
CpPAT10_0373	plcppse226	1x	PSE C
Cp1002_0813	plcppse227	1x	SECRETED
CpC231_0815	plcppse227	1x	SECRETED
cpfrc_00815	plcppse227	1x	SECRETED
CpI19_0815	plcppse227	1x	SECRETED
CpPAT10_0813	plcppse227	1x	PSE C
CpPAT10_0670	plcppse228	1x	PSE N
cpfrc_00669	plcppse228	1x	CYTOPLASMIC
CpI19_0669	plcppse228	1x	CYTOPLASMIC
Cp1002_0670	plcppse228	1x	CYTOPLASMIC
CpC231_0669	plcppse228	1x	CYTOPLASMIC
